# Highly Harsh-Environment-Stable and Sustainable Multifunctional Silk Textiles Enabled by Programmable Underwater Robust yet Stimulus-Reversible Cation–π Adhesion

**DOI:** 10.34133/research.0910

**Published:** 2025-10-03

**Authors:** Zi-Hao Wang, Fu-Rong Zeng, Jia-Yan Zhang, Rong Ding, Yan-Qin Wang, Bo-Wen Liu, Shu-Liang Li, Yu-Tao Wang, Xiu-Li Wang, Yu-Zhong Wang, Hai-Bo Zhao

**Affiliations:** ^1^ Collaborative Innovation Center for Eco-Friendly and Fire-Safety Polymeric Materials (MoE), State Key Laboratory of Polymer Materials Engineering, National Engineering Laboratory for Eco-Friendly Polymer Materials (Sichuan), College of Chemistry, Sichuan University, Chengdu 610064, P.R. China.; ^2^ Research Institute for Biomass Materials, Tianfu Yongxing Laboratory, Chengdu 610213, Sichuan, P.R. China.; ^3^ SINOPEC (Beijing) Research Institute of Chemical Industry Co., Ltd., Beijing, P.R. China.

## Abstract

Durable multifunctional textiles with advanced protective performance are increasingly crucial for maintaining stability in complex and harsh environments. However, the prevalent use of disposable synthetic surface treatments poses an intractable challenge to the circular economy focused on sustainability. In this study, we introduce an innovative biomimetic sustainable silk textile that is stable in harsh environments and offers high flame retardancy, antibacterial, and anti-mildew properties through a simple yet effective surface treatment strategy. This treatment leverages a specially designed aromatic polyorganosiloxane with quaternary ammonium structures, capable of programmable, robust, and stimulus-responsive reversible cation–π adhesion. The resulting strong water-insensitive cohesive energy from biomimetic cation–π interactions between aromatic and cationic moieties ensures that the multifunctional textiles exhibit excellent long-term durability, even under challenging conditions such as underwater, in saltwater, and in acidic or alkaline solutions. Moreover, the reversible reconstruction of cation–π adhesion allows for on-demand surface treatment recycling, achieving a 100% recycling rate. This study offers a new approach to creating smart and multifunctional textiles that combine sustainability with robust performance in harsh environments.

## Introduction

Textiles, as one of the earliest and most extensively integrated basic material formats into human daily lives, have been long viewed as the most fundamental form of personal protection [1]. As security threats grow more complex, there is a growing need for multifunctional textiles capable of withstanding damage from diverse harsh environments, including exposure to microorganisms, fire, friction, etc. [2,3]. Particularly pressing is the demand for durability in challenging conditions like underwater environments and exposure to salt, acid, and alkali solutions [2,4], where traditional functional textiles often fall short. Despite the progress, the current widespread durable multifunctional textiles mainly rely on permanent one-time chemical surface treatments, posing a continuous challenge to sustainable ecological development [5]. The release of microplastics during long-term application in harsh environments further highlights this issue [6]. By 2023, the persistence of microplastics from waste functional textiles had accounted for 35% of global primary microplastic pollution, necessitating urgent solutions [1]. Therefore, there is a pressing imperative to develop multifunctional textiles that combine durability in harsh environments with sustainability.

In recent years, various surface treatment strategies have emerged to enhance the durability and functionality of textiles in harsh environments. These include methods like covalent surface grafting [7], dye-fixing imitation [8], and superhydrophobic surface modifications [9]. For instance, flame-retardant and antimicrobial components can be covalently grafted onto textile surfaces or physically embedded into superhydrophobic coatings [10]. However, these chemical treatments are often irreversible and pose challenges for recycling, limiting their sustainability [11]. To address this, there is a growing interest in constructing reversible processes using noncovalent interactions such as electrostatic forces [12], supramolecular interactions [13], hydrogen bonding [14], and host–guest interactions [15], which markedly improve the material’s recyclability. Despite these advancements, the reversible noncovalent strategies universally suffer from poor adhesion, particularly in underwater and harsh environments [16], limiting their long-term effectiveness. Integrating robust harsh-environment durability with full recyclability in surface functional treatments still remains a major challenge. In addition, the existing methods often result in colors on surfaces [17], impacting the original appearance of the textile (including colors, patterns, and styles), which hinders their widespread adoption.

In nature, marine organisms, such as mussels, feature unique durable underwater adhesion [18,19]. Recent studies have uncovered that cation–π interactions, which are 3 times more energetic than coulomb interactions in water [20], play a key role in enhancing the adhesive and cohesive properties of mussel foot films [21]. Moreover, the latest research has focused on manufacturing durable flame-retardant or antibacterial textiles through cation–π adhesion surface treatment [22–24]. Therefore, by combining this understanding with the dynamic reversibility of noncovalent interactions [25], mimicking the mussel’s durable underwater adhesion mechanism through cation–π interactions serves as inspiration for the design of high-performance, sustainable, and multifunctional textiles.

**Fig. 1.**
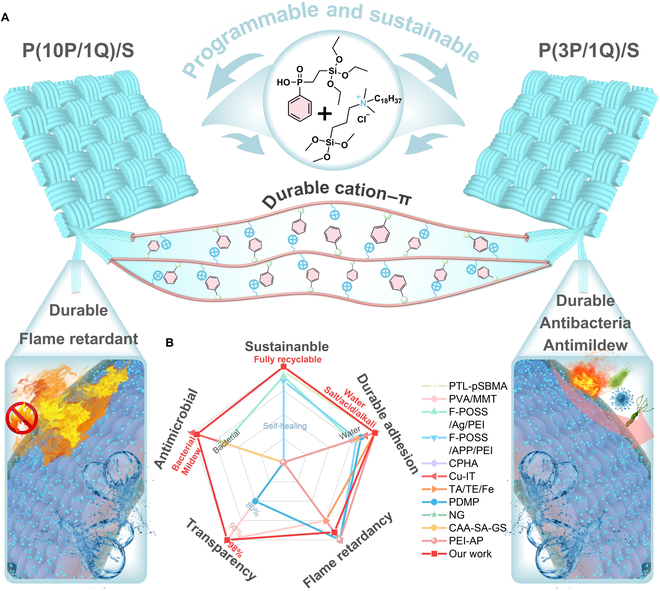
Structure and performance of multifunctional silk textiles. (A) Schematic illustrating the structure of the multifunctional silk textiles [P(PPA-Si/QAS)/S] and the performance of its flame retardancy, antibacterial and antimildew activity, durability, and sustainability. (B) Performance comparison of P(PPA-Si/QAS)/S with other reported multifunctional textiles.

Here, to address the dual challenges of integrating durability and recyclability from functional treatments, we present a new biomimetic strategy to create harsh-environment-stable and sustainable multifunctional textiles with excellent flame retardancy, antibacterial, and antimildew properties. We employ programmable, underwater, robust but stimulus-responsive reversible cation–π adhesion for high-performance surface treatments, while natural silk, as one of the most versatile and favorable fabrics, is chosen as the typical substrate of textiles. A specially designed multifunctional polyorganosiloxane, featuring P-doped aromatic and quaternary ammonium structures, named P(PPA-Si/QAS), is seamlessly deposited onto the surface of silk textiles without altering original colors (Δ*E*<2.5) and flexibility. Compared to other approaches, the abundance of aromatic and cationic moieties in our design allows for the formation of robust water-insensitive cation–π interactions, resulting in strong cohesive energy and durable adhesion throughout prolonged use. Remarkably, even when subjected to challenging conditions such as underwater environments, saltwater, or acidic/alkaline solutions (pH 1 to 14), our multifunctional silk textiles [P(PPA-Si/QAS)/S] exhibit exceptional stability over extended periods. Of particular interest is the ability of the durable cation–π interactions to be repeatedly disrupted and achieve 100% recycling through a simple ethanol infiltration–volatilization process. This programmable feature enables the surface modification treatments to be fully recyclable on demand, aligning with sustainable development goals (Fig. 1). This methodology offers a fresh perspective on the creation of smart and multifunctional textiles, combining sustainability with robust performance in harsh environments.

## Results

### Fabrication and characterization

The sustainable multifunctional silk textiles were fabricated via a simple and facile surface modification strategy starting with the synthesis of functional siloxane monomer (PPA-Si), followed by surface co-condensation with quaternary ammonium silane (QAS). In this design strategy, cross-linked polysiloxane serves as a durable and highly transparent backbone, providing superior stability in harsh environments [26]. The inclusion of phosphorus-containing aromatic pendant groups and QAS allows for tailored cation–π interactions [27], which enhance underwater adhesion and enable programmable functionalization, further strengthening the robustness of materials. As shown in Fig. S1, PPA-Si was first synthesized via a one-step addition action between triethoxyvinylsilane (VTES) and phenylphosphinic acid (PPA). Subsequently, the transparent precursor P(PPA-Si/QAS) with specific loading of QAS for programmable functions was obtained by hydrolysis condensation in an aqueous medium. Their chemical structure, confirmed by ^1^H nuclear magnetic resonance (NMR) and Fourier transform infrared (FTIR), is shown in Figs. S2 to S4. Notably, P(PPA-Si/QAS) can be repeatedly solved in ethanol to form transparent nanomicelles (~6.5 nm), due to a relatively limited degree of condensation. This, in turn, offers a versatile repurposing solution to the sustainable and programmable application (Fig. S5). Finally, multifunctional silk textiles were fabricated using a “dip-pad-cure” method with a specific P(PPA-Si/QAS). This method involves a further condensation reaction on the surface, allowing for sustainable reuse through completely reversible programmable cation–π interactions.

**Fig. 2.**
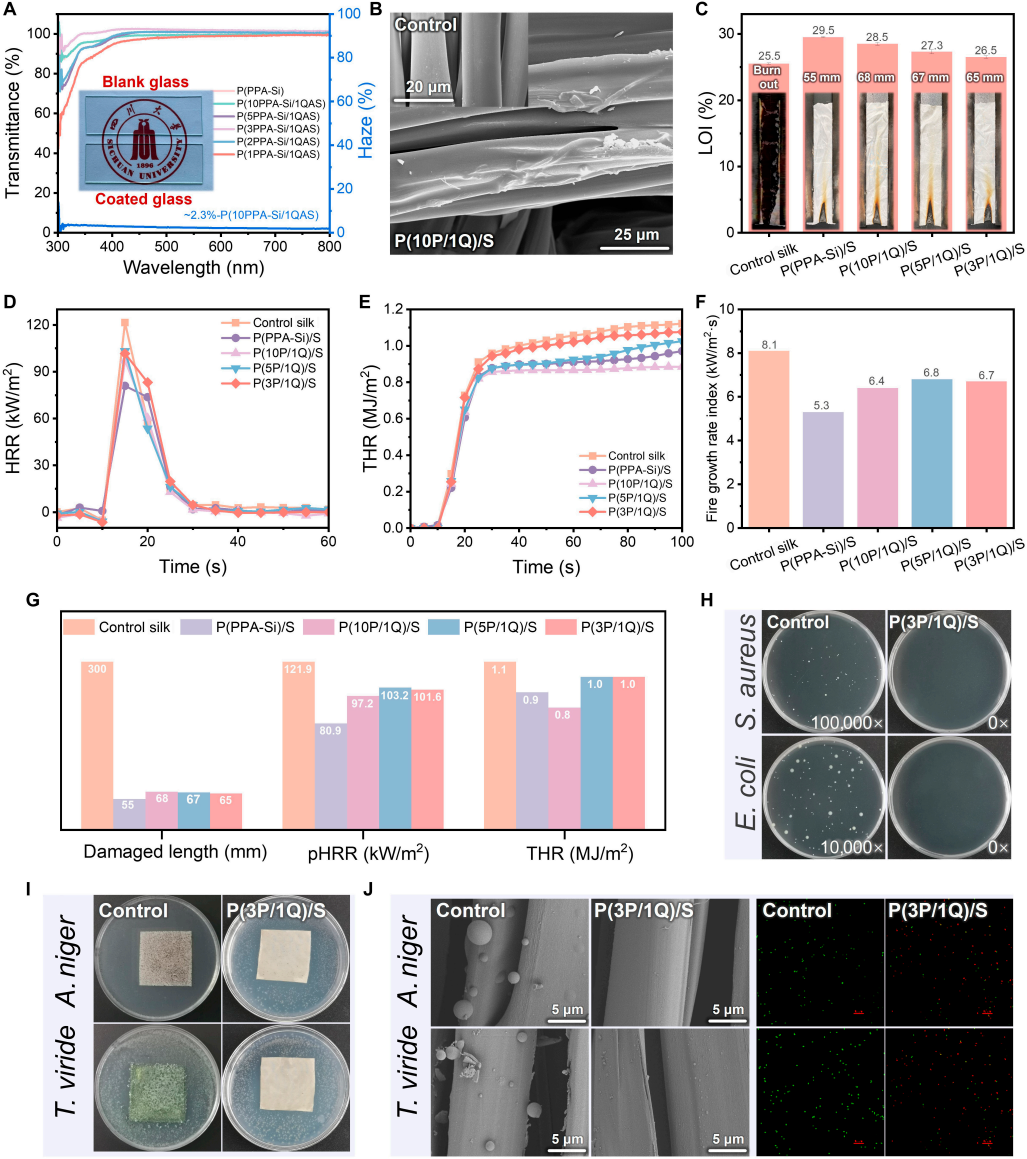
Characterization, flame retardancy, and antibacterial and antimildew properties of multifunctional silk textiles. (A) Transparency of P(PPA-Si) and P(PPA-Si/QAS). (B) SEM micrographs of control silk and P(10P/1Q)/S. (C) LOI values and digital photographs after the vertical flame test for the control silk, P(PPA-Si)/S, and P(PPA-Si/QAS)/S. (D to G) Heat release rate (HRR), total heat release (THR) curves, fire growth rate index (FIGRA), and flame retardancy comparison (damaged length, pHRR, and THR) of control silk, P(PPA-Si)/S, and P(PPA-Si/QAS)/S. (H) Typical photographs of antibacterial experimental results of control silk and P(3P/1Q)/S against *S. aureus* and *E. coli*. (I) Typical photographs and antimildew experimental results of control silk and P(3P/1Q)/S against *A. niger* and *T. viride*. (J) SEM images and confocal images of *A. niger* and *T. viride* treated with control silk and P(3P/1Q)/S, respectively. Note: green and red dots represent alive and dead *A. niger* or *T. viride*, respectively.

The resulting P(PPA-Si/QAS) exhibits colorless and high transparency regardless of the QAS loading, as demonstrated in visual inspection (Fig. 2A). When applied to glass surfaces, P(PPA-Si/QAS) achieves an almost uniform 98% to 100% optical transmittance and a low haze of only ~2.3%, surpassing previous reports [28]. This exceptional transparency ensures that P(PPA-Si/QAS) functionalized silk textiles maintain their original optical appearance without compromise. Large-area colored textiles treated with P(PPA-Si/QAS) retain vibrant colors and natural flexibility without any adverse effects, exhibiting characteristics similar to pristine textiles (Fig. S6). Quantitatively, the color coordinates (a*, b*) and lightness (L*) of the treated textile experience minimal changes, with a color difference (Δ*E*) as low as 2.5, imperceptible to the naked eye [29] (Fig. S7 and Table S3). For further fundamental and programmable research, we prepared various functionalized silk textiles by adjusting the ratio between PPA-Si and QAS. As shown in scanning electron microscopy (SEM) micrographs (Fig. 2B and Fig. S8), the control silk fibers show a clean and glossy surface morphology. After surface modification, all the functionalized silk textiles exhibit a similar rough fiber surface with a thin film layer wrapping around them, accompanied by some micro- and nanoparticle aggregation. Energy-dispersive spectroscopy (EDS) mapping images further confirm the well-preserved weave microstructures and the even distribution of the P(PPA-Si/QAS) film throughout the silk microfibers. The successful modification is also evidenced by distinct binding energy signals at 132.0 eV (P2p) and 101.3 eV (Si2p) in the x-ray photoelectron spectroscopy (XPS) results [30] (Fig. S9).

### Multifunctions of flame retardancy, antibacterial, and antimildew

P(PPA-Si/QAS) imparts excellent flame-retardant performance to flammable silk textiles. We evaluated the flame retardancy using the 2 most-used classic methods, namely, limiting oxygen index (LOI) and vertical flame test (VFT). As shown in Fig. 2C, the control silk textile displayed an LOI value of 25.5% and burned rapidly throughout the entire sample after ignited during the VFT. In contrast, the silk textile modified with P(PPA-Si) achieved a desired LOI value of 29.5% and exhibited self-extinguishing performance along with a considerably reduced residual char length (55 mm). Although the long alkyl chains in QAS are flammable, with the introduction of QAS in the treatment, the functionalized silk textiles were virtually incapable of catching fire. Even with the molar ratio of QAS reaching 1:3 for PPA-Si [P(3P/1Q)/S], the silk textile could still pass the VFT, indicating a satisfactory flame-retardant property (Fig. S10 and Table S4). Apart from small-scale functional textiles, we further evaluated the scalable preparation of functional silk textiles. As observed in Fig. S10G, the large-scale modified silk textile with a size of 110 cm × 60 cm was prepared successfully through a facile spraying process. As expected, the spray-modified large-size silk textile also demonstrated excellent flame retardancy (Fig. S10H), exhibiting great potential for large-scale applications. The flammability of the silk textile was further assessed by cone calorimetry, which reflects the burning behavior similar to the real fire scenario (Fig. 2D and E and Table S5). The control silk demonstrated a pronounced and elevated peak heat release rate (pHRR) of 121.9 kW/m^2^ and a total heat release (THR) value of 1.1 MJ/m^2^. Conversely, the silk textiles treated with P(PPA-Si/QAS) displayed a clear trend of inhibition, markedly reducing both pHRR and THR values. Correspondingly, the fire growth rate index (FIGRA), derived from the proportions of pHRR to the time to pHRR (*t*_pHRR_), exhibited a marked decline for all the treated silk textiles, demonstrating a substantial reduction in fire hazards (Fig. 2F). Following a quantitative comparison, the P(PPA-Si/QAS) treatment clearly substantially increased flame retardancy of silk textiles, which indicates remarkable flame-retardant protection, covering a range from small fire ignition to severe fire conditions (Fig. 2G) [31]. To unveil the flame-retardant mechanism, we analyzed residual char after combustion in detail. The functionalized silk textiles showed much thicker and higher yield residue char with a loose and numerous pore microstructure, which served as an insulation layer preventing the transfer of heat flux during combustion [32,33]. The lower *I*_D_/*I*_G_ values and the presence of additional elements of P and Si in the chemical composition further contribute to the efficacy of the P(PPA-Si/QAS) treatment, imparting exceptional condensed-phase barrier properties for flame retardancy (Figs. S11 to S14) [34].

Benefiting from the classical antibacterial and antifungal properties of the quaternary ammonium group in QAS, the functional silk textiles showed excellent microbial resistance, which was another highly desirable feature for protective function. A plate counting measurement was conducted to investigate the antibacterial activity against *Staphylococcus aureus* and *Escherichia coli*, as shown in Fig. 2H and Fig. S15. Following incubation, the culture medium treated with control silk displayed dense bacterial colonies, whereas the growth of the 2 bacteria was noticeably impeded beneath the P(3P/1Q)/S treatment. Consequently, the antibacterial rate of P(3P/1Q)/S reached more than 99.9999% for both *S. aureus* and *E. coli*, indicating an outstandingly high-efficiency antibacterial performance. For antimildew activity assessment, the common *Aspergillus niger* and *Trichoderma viride* were selected as model mildew. Remarkably, the colony formation on the P(3P/1Q)/S surface was substantially reduced compared to the control surface, resulting in an antimildew rate of 3 against *A. niger* and 1 against *T. viride* [35], respectively (Fig. 2I and Table S6). The further corroboration of the antimildew property was reflected in the micro-evaluation and live/dead fluorescence staining assays [36]. Compared with the pump and full morphology of microorganisms in the control group, no mold cells were observed in the P(3P/1Q)/S after incubation (Fig. 2J and Fig. S16). Similarly, almost all *A. niger* and *T. viride* treated with P(3P/1Q)/S were viewed in red color, which represents dead bacteria stained with red dye across the damaged cell membrane, revealing the disruption and lyse of most of the bacterial cells. Based on all of the analysis results discussed above, the functionalized silk textiles demonstrated excellent programmable multifunctionality of flame retardancy, antibacterial, and antimildew, making it a potential choice for relevant protective applications in specific environments. The integration of programmable flame retardancy and antimicrobial properties probably arises from a synergistic interplay between the P-doped aromatic structures and quaternary ammonium moieties in the polyorganosiloxane matrix. Combined with the flame-retardant mechanism, the P-containing aromatic groups not only enhance flame retardancy by promoting residue char formation but also stabilize the spatial distribution of QAS through robust cation–π interactions. Even at high temperatures or under harsh conditions, this dual-function architecture ensures that the QAS remains evenly anchored within the matrix, thereby maintaining sustained antimicrobial efficacy. This entrapment mechanism may further prolong antimicrobial activity, ensuring long-term microbial resistance during daily usage.

### Strong adhesion, durability, and harsh-environment stability

**Fig. 3.**
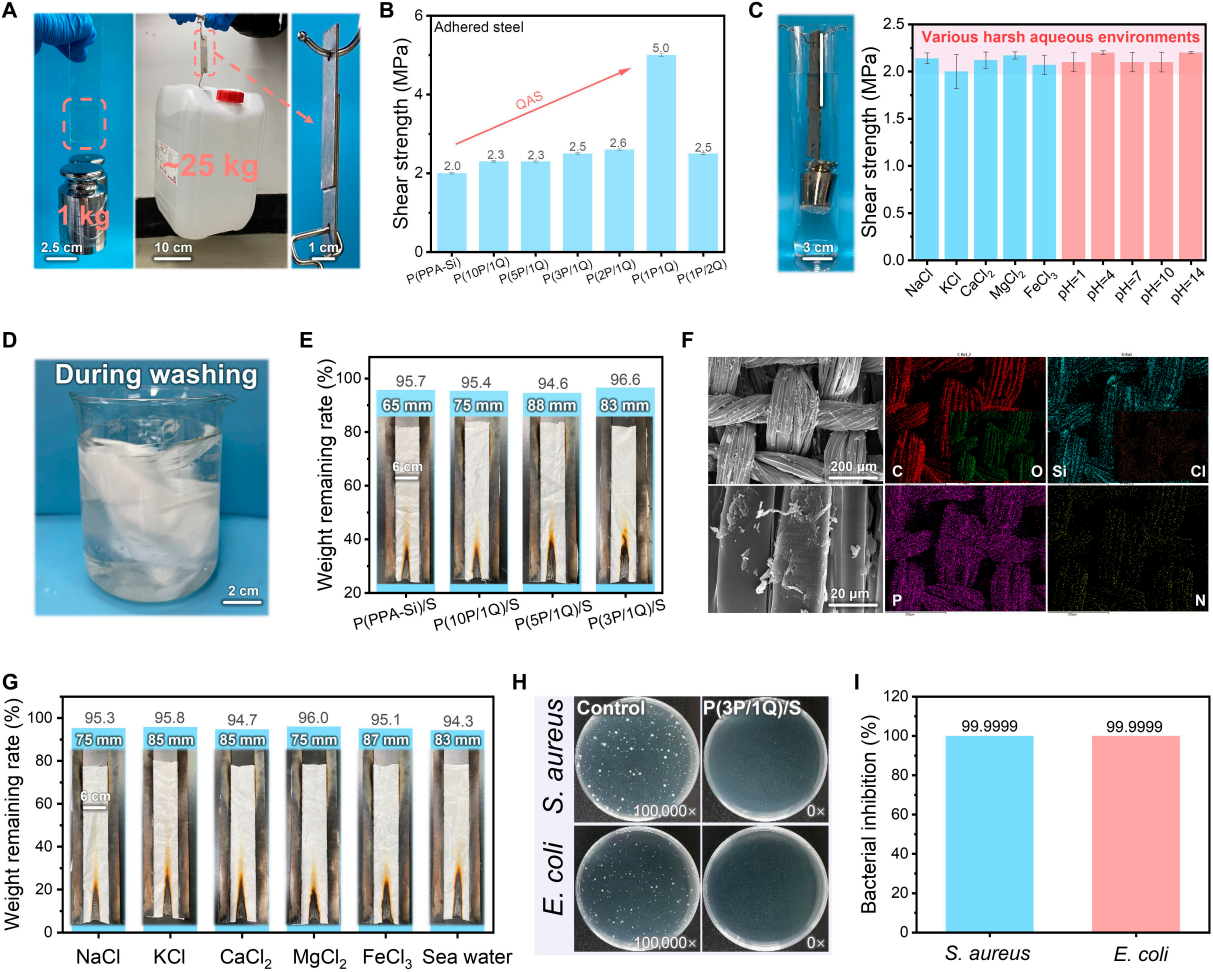
Durability and stability of multifunctional silk textiles under various harsh environments. (A) Photograph of P(10PPA-Si/QAS) adhered on glass and steel surfaces holding a 1-kg and a 25-kg weight loading, respectively. (B) Shear adhesion strength of P(PPA-Si) and P(PPA-Si/QAS) on steel substrate surface. (C) Adhesion stability of P(10PPA-Si/1QAS) after soaking in various pH values of 1 to 14 and different salt solutions (NaCl, KCl, CaCl_2_, MgCl_2_, and FeCl_3_) for 10 days. (D) Implementation method of durability against the water test for modified silk textiles. (E) LOI values and digital photographs after the vertical flame test for P(PPA-Si)/S and P(PPA-Si/QAS)/S after soaking in water for 10 days. (F) SEM micrographs and EDS mapping P(10P/1Q)/S after soaking in water for 10 days. (G) Digital photographs after the vertical flame test for the P(10P/1Q)/S after soaking in different salt solutions (NaCl, KCl, CaCl_2_, MgCl_2_, FeCl_3_, and seawater) for 10 days. (H and I) Typical photographs of antibacterial experimental results of control silk and P(3P/1Q)/S against *S. aureus* and *E. coli* after soaking in water for 10 days, respectively.

In the case of application durability, robust and durable adhesion between the interfaces is considered the most crucial determining factor. The interfacial adhesion performance to several substrates with relatively smooth surfaces was monitored by shear strength [14]. As depicted in Fig. 3A, P(PPA-Si/QAS) demonstrated exceptional “nonmarking” adhesion, capable of lifting a 25-kg weight with minimal contact area between the steel substrates. Such robust macroscopic adhesion is ascribed to strong intermolecular cohesive energy induced by cation–π interactions and van der Waals interactions on the interface [37]. In turn, serving as a verification, the adhesion strength of P(PPA-Si/QAS) stabilized at nearly 2.5 MPa and gradually increased with increasing molar ratio of QAS. When reaching a molar ratio of 1:1 between PPA-Si and QAS, the adhesion strength sharply jumped to 5.0 MPa, almost twice as high as that of other samples (Fig. 3B and Fig. S18). This phenomenon likely stemmed from the one-to-one correspondence pattern between the cation and phenyl ring, leading to the highest cation–π interactions compared with other samples. The cation–π interactions play a key role in enhancing the intermolecular cohesive energy and adhesive properties, resulting in the strongest intermolecular cohesive energy and a notable enhancement in adhesion strength [22]. The durability under water and various harsh environments is essential for the practical task-specific application. The adhesion strength of all P(PPA-Si/QAS) bonded to steel substrates remained relatively stable after soaking in water for 10 days. Even after 1 week of immersion in various harsh conditions (different salt and strong acid/alkali solutions), there was no pronounced decay in the adhesion strength, suggesting excellent durability. This excellent durability can be attributed to the robust water-insensitive cohesive energy from aromatic polyorganosiloxane driven by intermolecular cation–π interactions. The superior resistance to harsh conditions enables the P(PPA-Si/QAS) surface treatment to fully meet the requirement of long-term usage (Fig. 3C and Figs. S20 and S21).

As a desired result of the adhesion durability, the P(PPA-Si/QAS)/S shows excellent underwater and harsh-environment resistance. As observed in Fig. 3D and E, after being immersed in water for 10 days, the weight loading of P(PPA-Si/QAS) on the textile surface remained at a high level (around 95.0%) without apparent reduction regardless of the molar ratio between PPA-Si and QAS, further illustrating the water-resistant capability of cation–π adhesion. The chemical composition and surface morphology were almost identical after soaking in water (Fig. 3F and Fig. S22). Consequently, P(PPA-Si/QAS)/S displayed excellent durable water-resistant flame retardancy (Fig. 3E, Fig. S23, and Table S7). Even after treatment with various salt solutions, typically disrupting hydrogen bonding and electrostatic interactions, the flame retardancy of P(PPA-Si/QAS)/S demonstrated remarkable stability with minimal changes (Fig. 3G, Figs. S24 and S25, and Table S8). Furthermore, P(PPA-Si/QAS)/S also exhibited excellent stability with as high a weight remaining as 97.5% after 50 cycles of abrasion tests. The chemical structure, surface morphology, and flame retardancy of the textiles almost remained unchanged (Figs. S26 and S27 and Table S9). Correspondingly, the antibacterial activity of P(3P/1Q)/S immersed in water for 10 days showed an antibacterial rate of more than 99.9999% both against *S. aureus* and *E. coli*, which could ensure protection from microbial reproduction and contamination during daily use (Fig. 3H and I and Fig. S28). Overall, the strong cohesive energy driven by cation–π interactions guarantees enduring interfacial adhesion of P(PPA-Si/QAS), granting excellent resilience to a range of challenging environments. This quality is essential for functional textiles intended for use in complex settings.

### Mechanism for underwater robust cation–π adhesion

**Fig. 4.**
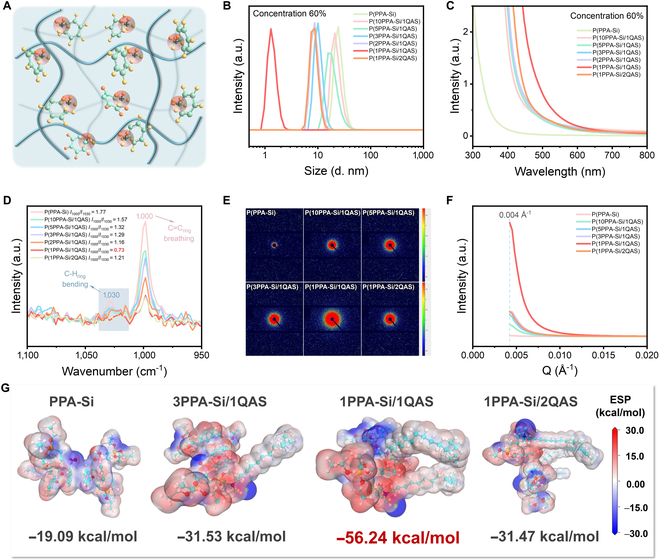
Mechanism for robust and durable cation–π adhesion. (A) Illustration of the cation–π interactions and robust cohesive energy within P(PPA-Si/QAS). (B and C) Particle sizes and UV–vis spectra of P(PPA-Si)/ethanol and P(PPA-Si/QAS)/ethanol solutions with a concentrate of 60%. (D) 785 nm excited resonance Raman spectra of P(PPA-Si) and P(PPA-Si/QAS). (E and F) 2D-SAXS patterns and 1D-SAXS integral curves for P(PPA-Si) and P(PPA-Si/QAS). (G) Energy distribution mappings for PPA-Si, 3PPA-Si/1QAS, 1PPA-Si/1QAS, and 1PPA-Si/2QAS through the density functional theory calculation.

The water-insensitive and robust cation–π adhesion is considered responsible for the strong cohesive energy, which further results in excellent durability and stability against various harsh environments (Fig. 4A). To unravel the mechanism behind cation–π interaction, we conducted characterization of micelle size and ultraviolet–visible (UV–vis) absorption of P(PPA-Si/QAS)/ethanol nanomicelles at various concentrations. All solutions exhibited a similar nanomicellar state with a particle size of around 6.0 to 7.0 nm at a low concentration of 15% (Fig. S29). The initial absorption wavelength gradually increased with the higher copolymerization molar content of QAS. However, an extreme anomalous phenomenon was observed when the concentration exceeded 60%. The nanomicelle diameter of P(PPA-Si/QAS) decreased steadily, which can be attributed to the formation of a smaller and denser aggregate structure [38]. This phenomenon is likely a result of the stronger cation–π interactions within the system. Eventually, the particle size of P(1PPA-Si/1QAS) nanomicelles plummeted to the lowest value at only 1.3 nm, while the maximum initial UV–vis absorption wavelength emerged at near 600 nm (Fig. 4B and C). These results demonstrated that the cation–π interactions promoted the sudden strongest intra-system cohesive energy when the cation and phenyl ring were in one-to-one correspondence patterns [39]. As a result, the viscosity of P(1PPA-Si/1QAS) nanomicelles was substantially enhanced compared to all other samples in dynamic rheology (Fig. S30). The strongest cation–π interactions were also confirmed by Raman spectroscopy, where the bands at around 1,000 and 1,030 cm^−1^ were attributed to the benzene breathing mode and out-of-plane bending of C-H ring, respectively. As depicted in Fig. 4D, the intensity ratio of *I*_1000_/*I*_1030_ decreased with the higher content of QAS until P(1PPA-Si/1QAS) reached its lowest value of 0.73. This decrease was probably associated with the marked enhancement of cation–π interactions [21,40]. We utilized small-angle x-ray scattering (SAXS) to validate the presence of cation–π interactions, which are typically characterized by discernible variations in electron cloud density [41], as evidenced by the SAXS pattern. As depicted in Fig. 4E and F, the scattering pattern of P(1PPA-Si/1QAS), featuring an equal molar ratio of PPA-Si to QAS, revealed a prominent scattering circle and the highest intensity of the principal scattering peak among the samples examined. This suggests a heightened level of microphase separation induced by cation–π interactions within this particular composition [41].

Further, the density functional theory (DFT) calculations were employed to investigate the interaction energies of the cation–π system [20]. Due to the complex cross-linked structures of poly(organosiloxanes), we chose the monomer of PPA-Si and QAS as molecular models to analyze the cation–π interactions (Fig. S31). As shown in Fig. 4G, for the system solely containing PPA-Si, where π–π interactions primarily prevail, the interaction energy amounted to −19.09 kcal/mol. With the addition of QAS and the creation of cation–π interactions (3PPA-Si/1QAS and 1PPA-Si/2QAS), the interaction energy decreased to nearly −31.5 kcal/mol, indicating stronger cohesion energy than that in pure PPA-Si. However, when the molar ratio reached 1:1, the interaction energy sharply dropped to −56.24 kcal/mol, a reduction of nearly 2 times. Combining the electrostatic potential results, it was found that the cation and phenyl ring in a one-to-one correspondence tended to bind tightly and drove the strongest cation–π interactions. In summary, the DFT calculation results highly coincide with the experimental results and further verify the crucial factor of cation–π interactions in achieving excellent, durable, and stable adhesion.

### Sustainable and programmable application

**Fig. 5.**
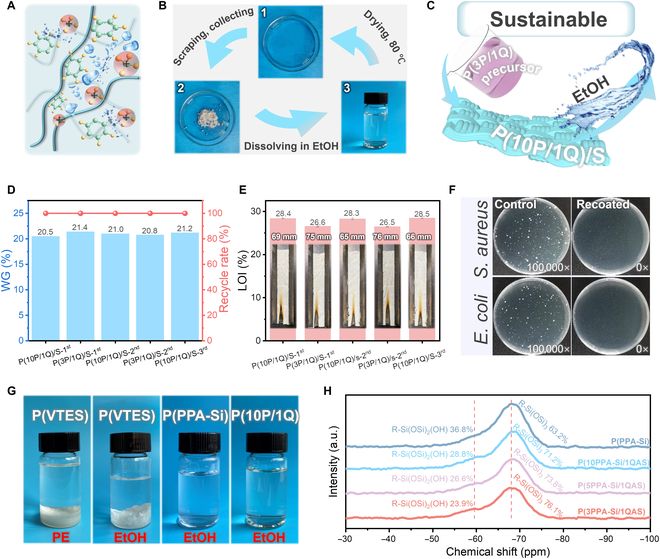
Programmable and sustainable application of multifunctional silk textiles and its mechanism. (A) Illustration of cation–π interactions destroyed by the ethanol infiltration. (B) Macroscopic process of the infiltration–volatilization cycle for P(10PPA-Si/1QAS) in ethanol. (C) Illustration of the sustainable and programmable application of P(PPA-Si/QAS). (D) Weight gain (WG) and recycle rate of P(PPA-Si/QAS) for programmable application at different reuse cycles. (E) LOI values and digital photographs after the vertical flame test for the recoated and programmable P(PPA-Si/QAS). (F) Typic photographs of antibacterial experimental results of recycled P(3P/1Q)/S against *S. aureus* and *E. coli*, respectively. (G) Dissolving behaviors of P(VTES), P(PPA-Si), and P(PPA-Si/QAS) in different organic solvents. (H) ^29^Si magic angle spinning NMR spectra of P(PPA-Si) and P(PPA-Si/QAS).

Embracing sustainable practices in the use of functional textiles offers a potent remedy for addressing escalating environmental concerns and reducing energy consumption during widespread commercial adoption [42]. Despite the exceptional adhesion durability in water and even harsh environments, P(PPA-Si/QAS) demonstrates the ability to repeatedly transform into nanomicelles in ethanol. This transformation occurs due to the reversible destruction of cation–π interactions (Fig. 5A and Fig. S32), suggesting that P(PPA-Si/QAS) holds promise for recyclability [43]. According to the findings presented in Fig. 5B, throughout the entire cyclic operation, transparent P(PPA-Si/QAS) nanomicelles, collected and infiltrated from the petri dish surface, could be fully reloaded onto the dish following ethanol drying. This process, coupled with the consistent adhesion performance even after 100 cycles of completely reversible adhesion on steel surfaces, indicates the capability of P(PPA-Si/QAS) to imbue functional textiles with remarkable programmability and application sustainability (Fig. S33).

Particularly highlighted in Fig. 5C, various functional silk textiles can be easily programmed through a straightforward infiltration–volatilization process in ethanol. Following immersion in excess ethanol and subsequent drying, the removal of P(10PPA-Si/1QAS) from the surfaces of functional silk textiles with high flame retardancy was facilitated, accompanied by the disruption of interfacial adhesion forces. The complete removal was further evidenced by SEM-EDS mapping images, which exhibited only C, O, and N elements with a clean and glossy surface morphology (Fig. S34A). Subsequently, another function, such as high antibacterial and antimildew properties, was reinstated by dipping the ethanol-treated silk textiles into corresponding P(3PPA-Si/1QAS) nanomicelles, following the original procedure. Following the full conversion process, the silk textiles displayed a similar surface morphology and chemical composition, indicating successful programmable refunctionalization (Fig. S34B). Even after 5 conversion cycles, maintaining a reloading weight of approximately 21.0 wt%, the system demonstrated an ideal recovery rate of up to 100% without any waste (Fig. 5D). Importantly, the re-achieved P(10P/1Q)/S exhibited the same excellent flame retardancy, boasting an LOI value of 28.4% and a steady residual char length of 67 mm. Similarly, the re-achieved P(3P/1Q)/S displayed comparable flame retardancy in VFTs and maintained antibacterial activity, with no observed bacterial colonies (Fig. 5E and F, Figs. S35 and S36, and Table S10). Overall, the P(PPA-Si/QAS) surface functionalization treatment seamlessly integrates durability and recyclability with a multifunctional design featuring high transparency, flame retardancy, antibacterial, and antimildew properties. By comparing the comprehensive performance with recent reported functional textiles, this approach demonstrates great advantages in simultaneously achieving functional durability and sustainability [2,44–47]. Notably, this strategy provides a highly valuable insight into manufacturing multifunctional textiles with harsh-environment stability and full sustainability, which can address key challenges in practical applications and aligns perfectly with the strategic goals of achieving carbon peak and carbon neutrality (Fig. 1B and Table S2).

The unique and freely programmable recyclable application in ethanol is speculated to be closely correlated with 2 key aspects: firstly, the repeatable solubility of water-insensitive P(PPA-Si/QAS) in ethanol, and secondly, the regeneration of strong cohesive energy during ethanol volatilization. On the one hand, although 3-dimensional cross-linked poly(organosiloxane) was typically recognized as insoluble in most organic solvents, P(PPA-Si/QAS) prepared in this work exhibited high solubility in ethanol. As shown in Fig. 5G, the unmodified P(VTES) without large pendant groups was transformed into precipitate in organic solvents such as petroleum ether and ethanol. However, after the covalent introduction of PPA and subsequent copolymerization with QAS, it directly became completely compatible with ethanol and dissolved into a transparent solution. This interesting behavior was probably associated with the reduced degree of condensation, which was limited by the large steric hindrance of the pendant group PPA. As confirmed by cross-linked structure analysis using solid-state ^29^Si NMR measurements, the ratio of R-Si(OSi)_2_(OH) to R-Si(OSi)_3_ for P(PPA-Si) and P(PPA-Si/QAS) was markedly higher compared to that of P(VTES), indicating that a large number of Si-OH groups did not participate in the condensation process [25] (Fig. 5H and Fig. S37). This led to the unique ideal solubility observed.

On the other hand, the regeneration of strong cohesive energy induced by cation–π interactions during ethanol volatilization was identified as another key factor for recyclable and sustainable applications. To illustrate this, the ethanol volatilization process was simulated by varying concentrations of P(PPA-Si/QAS) within the range of 10% to 80% [taking P(10PPA-Si/1QAS) as an example, Fig. S38]. Below a concentration of 60%, all solutions exhibited a clear transparent state, with nanomicelles steadily increasing in size from 2.7 to 9.2 nm as concentrations rose (Fig. S39A). However, the solutions gradually became less flowable, indicating a substantial enhancement in cohesive energy driven by cation–π interactions. Correspondingly, higher macroscopic viscosity at relatively higher concentrations further confirmed the stronger cohesive energy during ethanol volatilization (Fig. S39B). These findings highlight that cation–π interactions, realized through ingenious structural design, contribute to both recyclable sustainable applications and excellent resistance to underwater and harsh environments.

## Discussion

To summarize, in response to the challenges of environmental pollution from disposable textiles, we have shown a novel biomimetic surface treatment strategy for creating high-performance, harsh-environment-stable, and sustainable multifunctional silk textiles utilizing programmable underwater robust yet stimulus-responsive reversible cation–π adhesion. This strategy features the incorporation of specially designed P(PPA-Si/QAS) containing P-doped aromatic and quaternary ammonium structures. The resultant P(PPA-Si/QAS) effectively endows silk textiles with excellent flame retardancy, antibacterial (99.9999%), and antimildew activity, all without leaving any discernible marks on the surface. Driven by the cation–π interactions from the abundant side aromatic and cation groups, P(PPA-Si/QAS) exhibits strong cohesive energy, resulting in durable interfacial adhesion (up to 5.0 MPa shear strength on steel) during long-term application. Even after being subjected to complex, harsh conditions of underwater, saltwater, and acidic/alkaline solutions (pH 1 to 14), the multifunctional silk textiles emerge exceptional stability (with a weight retention of 95%), where the flame retardancy, antibacterial, and antimildew activity can be maintained without decay. Beyond the durability, by applying the infiltration–volatilization process of ethanol, the cation–π interactions can be completely disrupted and repeatedly rebuilt on demand, indicating a full recyclability with a 100% recovery rate of the surface modification treatments. The corresponding mechanism analysis, grounded in rigorous experimental results and theoretical simulation calculations, validates that stimulus-responsive cation–π interactions contribute to the full recyclability and durable adhesion. This methodology provides a renewed insight into the development of multifunctional integrated textiles, emphasizing both sustainability and harsh-environment stability, advancing toward a circular life-cycle future.

## Materials and Methods

### Materials

Silk textiles (70 g/m^2^) were supplied by Shaoxing Manheng textile sales company, China. PPA, VTES, and dimethyloctadecyl [3-(trimethoxysilyl) propyl] ammonium chloride (QAS) was purchased from Shanghai Aladdin Biochemical Technology Co., Ltd., China. Sodium hydroxide (NaOH) was obtained from Tianjin Beichenfangzheng Chemical Co., Ltd., China. Ethanol (AR), trichloromethane (TCM), petroleum ether (PE), sodium chloride (NaCl), potassium chloride (KCl), calcium chloride (CaCl_2_), magnesium chloride (MgCl_2_), and ferric chloride (FeCl_3_) were supplied by Chengdu Kelong Chemical Co., Ltd., China. All materials were used without further treatment.

### Synthesis of PPA-Si monomer and P(PPA-Si/QAS) precursor

PPA-Si was synthesized via the addition reaction between PPA and VTES. In detail, PPA (28.4 g, 0.2 mol) and VTES (39.9 g, 0.2 mol) were added to a 500-ml round-bottomed flask containing 50 ml of trichloromethane. After the reactants were dissolved completely, the mixture solution was heated to 80 °C, and the reaction was kept for 24 h under an N_2_ atmosphere. Then, after removing the solvent by rotary evaporation, the colorless and transparent viscous PPA-Si was obtained with overall yields of >90%.

P(PPA-Si/QAS) precursor was prepared by the co-hydrolysis–condensation reaction between PPA-Si and QAS in a mixture medium of ethanol/water (V/V, 2:1). Briefly, PPA-Si and QAS in a certain molar ratio were dissolved in the mixture solvent with a concentration of 20% and reacted for 8 h while stirring at 80 °C. After the reaction, the homogeneous and transparent P(PPA-Si/QAS) precursor was obtained for the surface modification of silk textiles. By adjusting the loading amount of QAS (molar ratio PPA-Si:QAS = 10:1, 5:1, 3:1, 2:1, 1:1, and 1:2), a different P(PPA-Si/QAS) precursor was obtained for comparison study, which were correspondingly named P(10PPA-Si/1QAS), P(5PPA-Si/1QAS), P(3PPA-Si/1QAS), P(2PPA-Si/1QAS), P(1PPA-Si/1QAS), and P(1PPA-Si/2QAS). In addition, the P(PPA-Si) precursor was also prepared following the same procedure as for the preparation of P(PPA-Si/QAS) precursor, but without introducing QAS.

### Fabrication of multifunctional silk textiles modified with P(PPA-Si/QAS)

The multifunctional silk textiles were prepared through a facial dip-coating process. In detail, based on the material-to-liquid ratio of 1:20, the control silk textile was first dipped into a given P(PPA-Si/QAS) precursor at 60 °C for 1 h and completely dried at 80 °C for further co-condensation reactions on the fabric surface. Until the weight was unchanged, the multifunctional silk textile, named P(PPA-Si/QAS)/S, was achieved. According to this dip-coating produce, different P(PPA-Si/QAS)/S were obtained by changing the precursor, which were named P(PPA-Si)/S, P(10P/1Q)/S, P(5P/1Q)/S, P(3P/1Q)/S, and P(1P/1Q)/S, respectively. The weight gain (WG) was calculated by the following formula: WG (%) = (*w*_1_ − *w*_0_)/*w*_0_ × 100%, where *w*_0_ represents the weight of the control silk textiles and *w*_1_ represents the weight of the modified textiles. Here, due to the same concentrations of different precursors, the WGs of all the modified silk textiles were around 20% (Table S1).

The spraying process is performed to fabricate large-scale functional silk textiles. Taking P(10P/1Q)/S as an example, the P(10PPA-Si/1QAS) precursor was first transferred into a spray bottle. Then, a uniform double-sided spraying process was conducted from a height of about 20 cm above the silk textile with a size of 110 cm × 60 cm. By regulating the amount of spraying, the coating weight loading was controlled to about 20 wt%. After complete drying at 80 °C, the large-scale functional silk textile was obtained and named LP(10P/1Q)/S.

### General characterization

FTIR spectra were recorded on a Nicolet 6700 instrument (Thermo Fisher Scientific Co., USA) with a wavenumber ranging from 400 to 4,000 cm^−1^. ^1^H NMR spectra were conducted on a Bruker AV II-400 MHz nuclear magnetic resonance instrument (Bruker, Switzerland) using dimethyl sulfoxide as the solvent. Solid-state ^29^Si NMR investigation of poly(organosiloxane) was carried out on a Bruker-600 NMR 600 MHz spectrometer (Avance, Bruker, Switzerland). The peak Q^1^, Q^2^, and Q^3^ correspond to (R-Si(OSi)(OH)_2_), (R-Si(OSi)_2_(OH)), and (R-Si(OSi)_3_), respectively. The degree of condensation (DC) was calculated from the peak areas of Q^1^, Q^2^, and Q^3^ based on the following equation: DC = (Q^1^ + 2Q^2^ + 3Q^3^)/3.

SEM images were taken on a JSM-7500F (JEOL) with an accelerating voltage of 15 kV. SEM was equipped with an energy-dispersive x-ray spectrometer to analyze element compositions and distribution maps. UV–vis spectra were obtained on a UV1800 UV–vis scanning spectrophotometer (Shimadzu, Japan). The color difference was recorded using an NR10QC colorimeter. Micelle size was measured on a Zetasizer Nano ZS (Malvern). The SAXS experiments were performed on a Xeuss 2.0 diffractometer. The shear adhesion strength was tested by using an electronic universal material testing machine (Instron 3366, USA). The LOI was measured on a HC-2C oxygen index instrument (Jiangning, China) with specimen dimensions of 160 mm × 100 mm according to the standard method DIN EN ISO 15025. VFT was carried out using a CZF-3 instrument (Jiangning, China) according to the GB/T 5455-1997 standard method, and the specimen dimension of the sample was 300 mm × 80 mm. The cone calorimeter test was conducted by a cone calorimeter under a heat flux of 35 kW/m^2^, according to the ISO 5660-1 standard. The specimens used for the test were 100 mm × 100 mm × 0.2 mm. Rheological measurements were performed on a Discovery HR-2 (TA Instrument, USA) within the linear viscoelastic region of 5% at a fixed temperature of 25 °C. Thermogravimetry-infrared spectrometry (TG-IR) was measured using a TA TGA5500 thermogravimetric analyzer linked with a Nicolet iS50 FT-IR spectrophotometer, which was performed at a 20 °C/min heating rate from 40 to 700 °C in a nitrogen atmosphere (flow rate: 25 ml/min). Raman spectroscopy was carried out using a laser Raman spectrometer (LabRAM HR, HORIBA Co., France) in the range of 800 to 2,000 cm^−1^ with an excitation wavelength of 532.17 nm. XPS was performed on an XSAM 800 spectrometer (KRATOS, UK) using Al Ka excitation radiation (1,486.6 eV).

## Data Availability

All data needed to evaluate the conclusions in the paper are present in the paper and/or the Supplementary Materials. Additional data related to this paper may be requested from the authors.
