# Supplementary material for: Highly Harsh-Environment-Stable and Sustainable Multifunctional Silk Textiles Enabled by Programmable Underwater Robust yet Stimulus-Reversible Cation–π Adhesion
Source: Research (Wash D C). 2025 Oct 3;8:0910. doi: 10.34133/research.0910 (PMC12491790; doi:10.34133/research.0910)
Supplement: Supplementary 1 — Figs. S1 to S39 Movies S1 to S5 [file research.0910.f1.docx]

Supplementary Materials for

**Highly Harsh-Environment-Stable and Sustainable Multifunctional Silk Textiles Enabled by Programmable Underwater Robust yet Stimulus-Reversible Cation-π Adhesion**

Zi-Hao Wang^1, 2^, Fu-Rong Zeng^1^, Jia-Yan Zhang^1^, Rong Ding^1^, Yan-Qin Wang^1^, Bo-Wen Liu^1^, Shu-Liang Li^3^, Yu-Tao Wang^3^, Xiu-Li Wang^1^*, Yu-Zhong Wang^1^, Hai-Bo Zhao^1^*

^1^ Collaborative Innovation Center for Eco-Friendly and Fire-Safety Polymeric Materials (MoE), State Key Laboratory of Polymer Materials Engineering, National Engineering Laboratory for Eco-Friendly Polymer Materials (Sichuan), College of Chemistry, Sichuan University, Chengdu, 610064, P.R. China

^2^ Research Institute for Biomass Materials, Tianfu Yongxing Laboratory, Chengdu 610213, Sichuan Province, P.R. China

^3^ SINOPEC (Beijing) Research Institute of Chemical Industry Co., Ltd.

*Corresponding author. E-mail: [haibor7@163.com](mailto:haibor7@163.com); [xiuliwang1@163.com](mailto:xiuliwang1@163.com)

**This supplementary information file includes:**

Supplementary methods and texts

Supplementary Figures S1 to S39

Supplementary Tables S1 to S10

Supplementary References 1 to 17

**Other Supplementary Materials for this manuscript include the following:**

Supplementary movies S1 to S5

Supplementary Methods

**Recycling-Reusing Process of P(PPA-Si/QAS) on the Surface of Silk Textile for Sustainable Application**

To evaluate the recycle process of P(PPA-Si/QAS), the modified silk textiles were soaked in excess ethanol until the P(PPA-Si/QAS) was removed completely and then extracted followed by fully drying at 80 ℃. Subsequently, after removing the ethanol by evaporating, the P(PPA-Si/QAS) was collected and redissolved into ethanol to prepare precursor with a concentration of 20% for further surface modification according to the original produce.

**Preparation of Adhesion Samples and Lap Shear Tests**

For adhesion application, most of the solvent in the given P(PPA-Si/QAS) precursor was first evaporated. Then, the given P(PPA-Si/QAS) was deposited on the surface of two substrates to be adhered with an area of 1 cm × 2 cm. Subsequently, the adhesive area of two substrates was pressed at a force of around 2 N. After complete drying and the adhesive set, the adhered substrates can be directly used for lap adhesion strength. According to the standard ASTM F2255, the lap adhesion shear tests were performed using a universal testing machine at a strain rate of 5 mm/min.

**Recycling-Reusing Adhesion Methods for P(10PPA-Si/1QAS) on Steel Surfaces**

First, a quantity of P(PPA-Si/QAS) was adhered to the surface of steel substrate. When recovered, the P(PPA-Si/QAS) was removed from the surface simply by rinsing and dissolving with excess ethanol to form a homogeneous solution. Then, after the most ethanol was evaporated, the recycled P(PPA-Si/QAS) precursor was obtained for the adhesion application.

**Durability Tests**

To assess the durability against water and harsh conditions, the modified silk textiles were soaked in water and various salt and acid solutions for several days. After that, the textiles were extracted and dried at 80 ℃ for 4 h for further study.

An abrasion resistance test was also performed to evaluate the durability of the P(PPA-Si/QAS) modified silk textiles. In detail, with a loading of 500 g weight, a piece of sandpaper was placed on the modified silk textiles and moved over the entire length along the lengthwise direction. A back and forth was recorded as 1 cycle of abrasion. After 50 cycles, the textiles were turned over, and another 50 cycles of abrasion were completed.

**Antibacterial activity**

According to the GB/T 20944.3-2008 and GB 4789.2-2016 test methods, we evaluated the antibacterial performance of the textile samples against *Staphylococcus aureus* (*S. aureus*, ATCC 6538), *Escherichia coli* (*E. coli*, ATCC 8739), *Aspergillus niger* (*As p. niger* ATCC, 16404) and *Trichoderma viride* (*T. viride*, ATCC, 26802), respectively. First, a bacterial suspension with a concentration of 10^6^ CFU/ml was prepared, then a textile sample (2×2 cm^2^) was immersed in 10 ml bacterial suspension for 6 h at 37 ℃ with constant shaking (200 rpm). After that, 1 ml of the treated bacterial suspension was taken out and added to a sterile petri dish with LB solid medium. Subsequently, the colonies were counted by dilution after incubation for 18 h at 37 ℃. The bacterial inhibition (R) was calculated by the following equation:

$$R\left( \% \right)=\frac{N_{c}-N_{t}}{N_{c}}\times100\%$$

Where *N_c_* and *N_t_* represent the number of colonies of the surviving bacterial cells in blank control and test textile samples, respectively.

**Antimildew tests**

The antimildew activity of textile samples was evaluated according the GB/T 174-2007 plating method. In brief, the modified textile sample (P(3P/1Q)/S) was placed into PDA medium, followed by a 1 ml of *Aspergillus niger* (*As p. niger* ATCC, 16404) or *Trichoderma viride* (*T. viride*, ATCC, 26802) suspension dropping. Then, the plate was shaken lightly until the mildew suspension was evenly distributed. After incubation at 28 ℃ for 28 days, the growth of mildew on the textile surface was rated. The rating standard is as follows:

0 rating: no obvious mildew was observed under a magnification of 50 times.

1 rating: Mildew is not visible to the naked eye or is difficult to see, but it becomes obvious under magnification.

2 rating: Mildew is easily noticeable to the naked eye, covering an area of 10%~30% on the surface of the sample.

3 rating: Mildew is easily noticeable to the naked eye, covering an area of 30%~60% on the surface of the sample.

4 rating: Mildew is easily noticeable to the naked eye, covering an area of more than 60% on the surface of the sample.

**The morphology observation and fluorescence staining assays of live/dead bacterial**

Morphology observation: the textile sample after culturing with mildew was washed with a buffer solution (PBS) for three times and fixed with a 10-fold volume of electron microscopy fixative. The fixed sample was rinsed three times, for 15 min each time, with a 0.1 M phosphate buffer solution (PB) at pH of 7.4. Subsequently, the sample was dehydrated using ethanol, dried, sputter-coated with gold and observed by scanning electron microscopy (5 kV, S-3400N).

Fluorescence staining assays: the textile sample after culturing with mildew was repeatedly washed with 1 ml of PBS buffer solution to remove the spores on surface. The spores were stained using the Calcein SG/PI Cell Viability and Cytotoxicity Assay Kit (BioVision, Catalog Number: C2015M). The intact spores were stained with cell-permeant SYBR Green (SG) dye, dead bacteria were re-stained with disodium iodide (PI) red dye (a cationic fluorescent dye). After incubating at 37 ℃ in a light-protected environment for 30 min, imaging was performed using a super-resolution confocal microscope (Nikon N-STORM).

**Density Functional Theory (DFT) Calculations**

All DFT calculations were carried out using the CP2K code. All calculations employed a mixed Gaussian and planewave basis sets. Core electrons were represented with norm-conserving Goedecker-Teter-Hutter pseudopotentials, and the valence electron wavefunction was expanded in a double-zeta basis set with polarization functions along with an auxiliary plane wave basis set with an energy cutoff of 400 eV. The generalized gradient approximation exchange-correlation functional of Perdew, Burke, and Enzerhof (PBE) was used. Each configuration was optimized with the Broyden-Fletcher-Goldfarb-Shanno (BGFS) algorithm with SCF convergence criteria of 1.0×10^-6^ au. To compensate the long-range van der Waals dispersion interaction, the DFT-D3 scheme with an empirical damped potential term was added into the energies obtained from exchange-correlation functional in all calculations.

Supplementary Figures and Texts


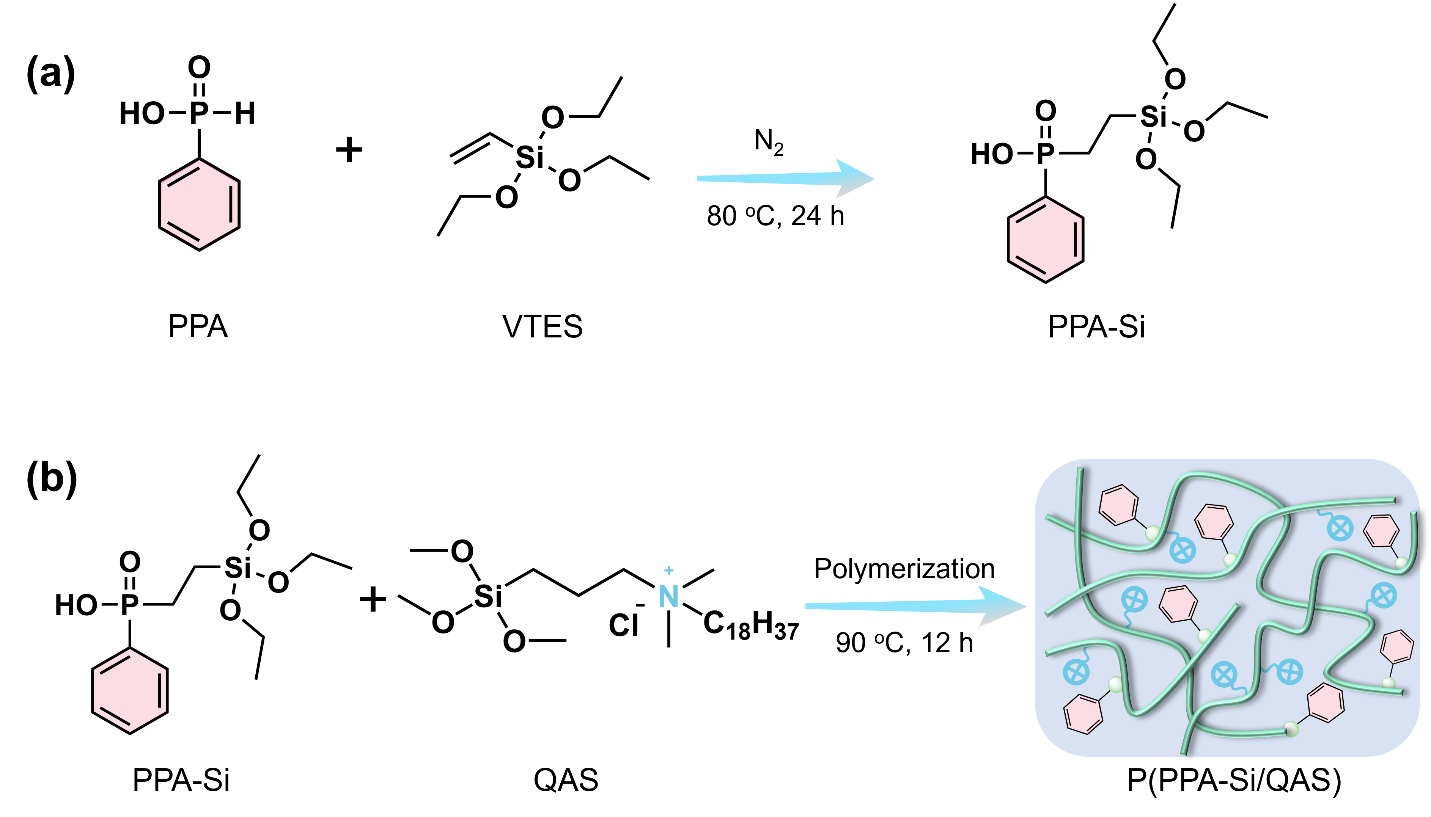


Fig. S1. Synthesis of PPA-Si and P(PPA-Si/QAS). Synthesis of P(PPA-Si) and P(PPA-Si/QAS).


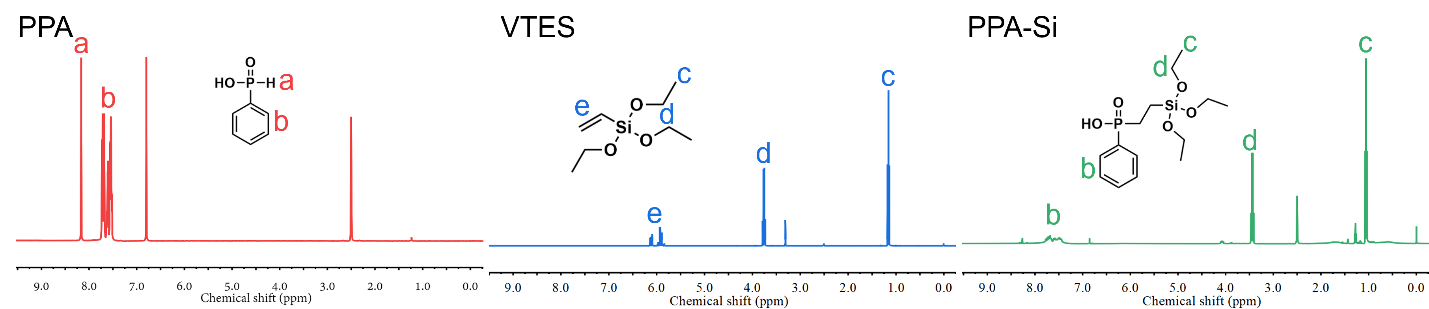


Fig. S2. ^1^H NMR characterization. ^1^H NMR spectra of the reactants and PPA-Si.

As shown in **Fig. S2**, the chemical structure of PPA-Si was characterized using ^1^H NMR. For the raw materials PPA and VTES, the characteristic absorption peaks at 8.47 ppm and 5.90-6.21 ppm in the ^1^H NMR spectrum were attributed to the P-H bond and C=C, respectively. However, in the spectrum of PPA-Si, the absorption peaks corresponding to P-H and C=C almost disappeared. Instead, two new characteristic absorption peaks corresponding to Si-CH_2_ and P-CH_2_ at 2.30 ppm and 0.85 ppm appeared, indicating the successful addition of P-H to C=C.


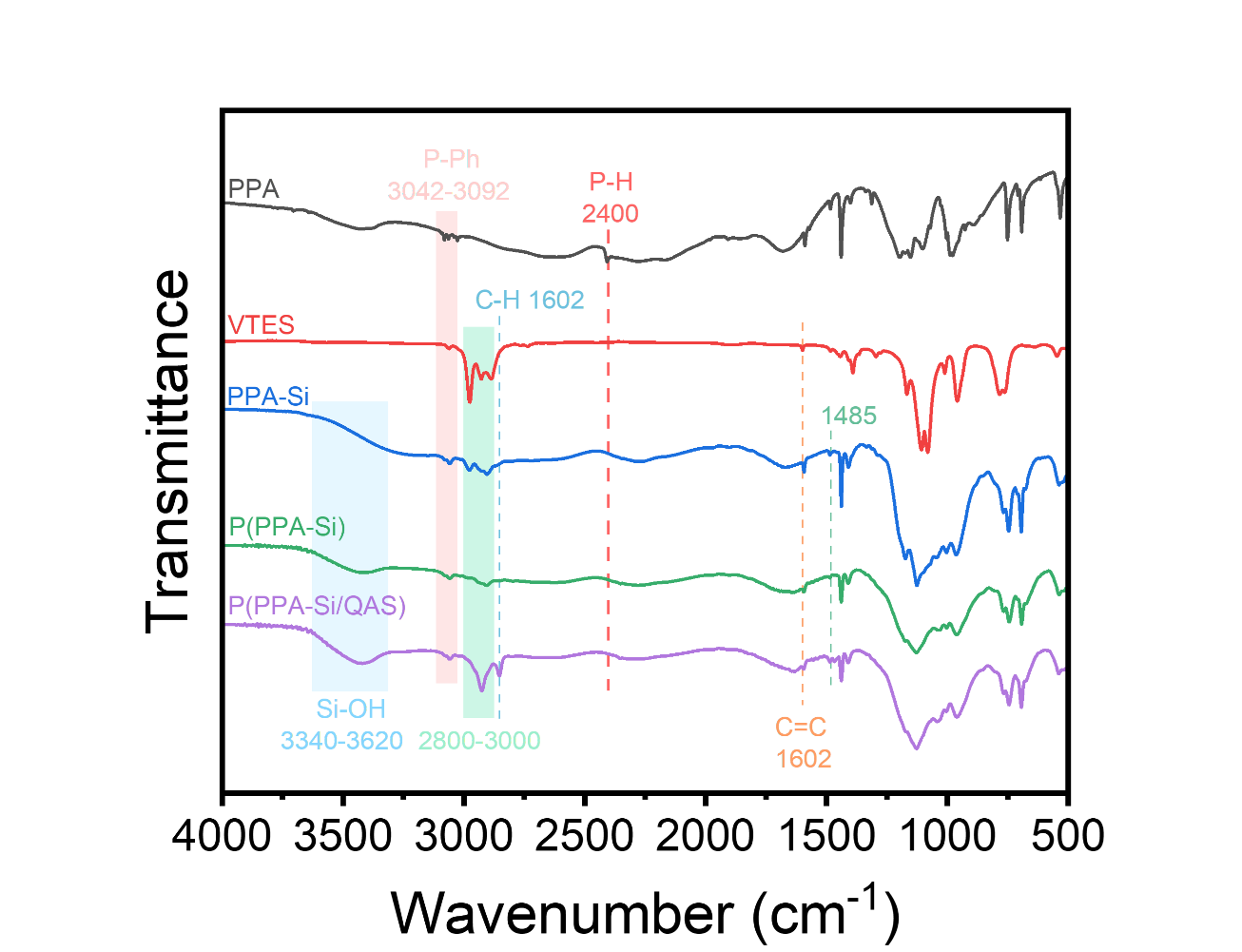


Fig. S3. FT-IR characterization. FT-IR spectra of reactants and PPA-Si.

The successful preparation of PPA-Si, P(PPA-Si), and P(PPA-Si/QAS) was verified by FTIR. For the PPA-Si spectrum, the characteristic absorption peaks corresponding to P-H and C=C at 2314 cm^-1^ and 1600 cm^-1^, respectively, disappeared, suggesting the successful addition of VTES and PPA. After the condensation of PPA-Si, new absorption peaks appeared in the range of 3340-3620 cm^-1^, which can be attributed to the Si-OH absorption peak. The formation of this absorption peak may be ascribed to the greater steric hindrance of PPA, which restricted the complete condensation of the ethoxide groups after hydrolysis, resulting in residual Si-OH groups. After introducing QAS for copolymerization, there was a distinct absorption peak at 1602 cm^-1^ corresponding to the methylene groups on the long alkyl chain of QAS, indicating the successful preparation of P(PPA-Si/QAS).


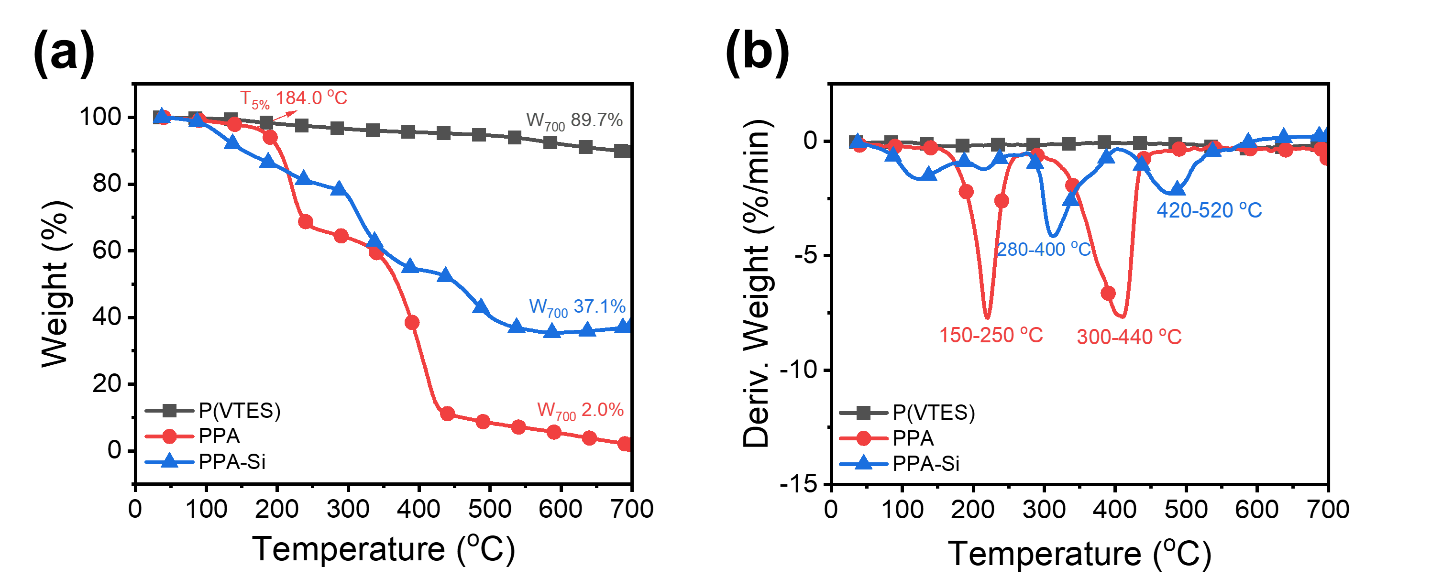


Fig. S4. Thermal decomposition behavior. (a) TG and (b) DTG curves of the reactants P(VTES) and PPA-Si.

Compared to that of the raw materials, the thermal stability of the PPA-Si product after the covalent addition reaction changed significantly. As shown in **Fig. S4**, P(VTES) exhibited excellent thermal stability, while PPA showed poor thermal stability and started to degrade at 184 °C. PPA-Si underwent a two-step degradation process mainly between 280-400 ℃ and 420-520 ℃. Although it showed a lower initial degradation temperature, the residual char yield at high temperatures was significantly greater than that of PPA (37.1%).


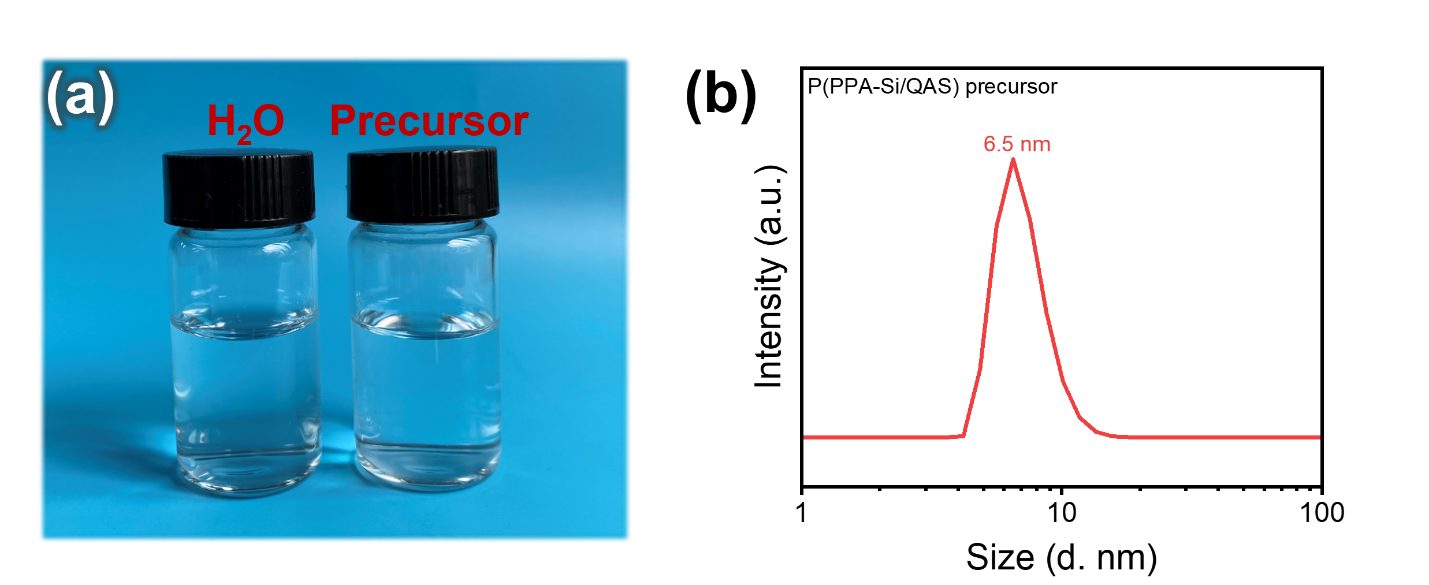


Fig. S5. Dissolution behavior of P(PPA-Si/QAS) in ethanol. (a) Digital photographs of water and the P(PPA-Si/QAS) precursor. (b) Particle size of nanomicelles in the P(PPA-Si/QAS) precursor.


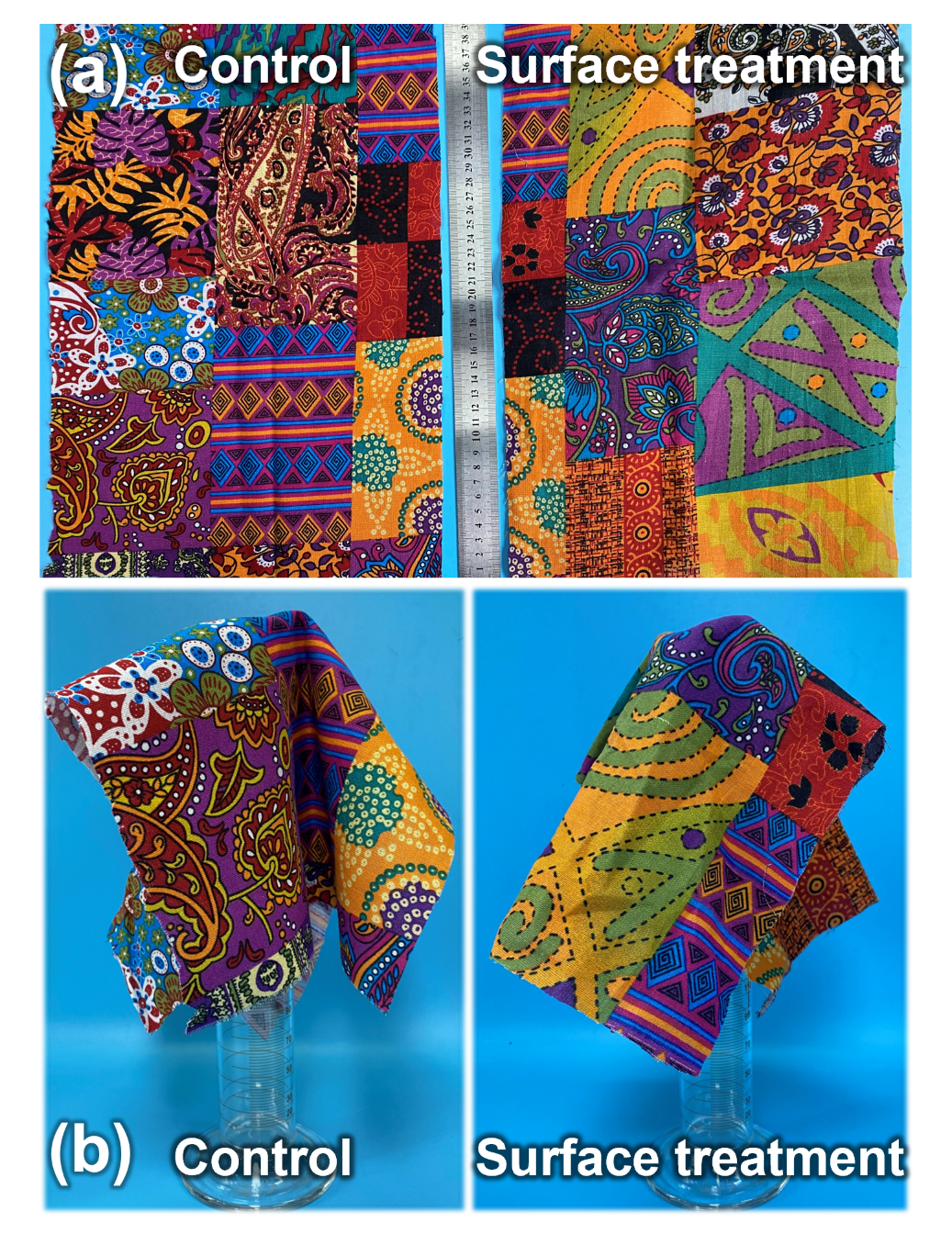


Fig. S6. Appearance of control and surface treated silk. (a) Colors and patterns and (b) flexibility of the control silk and P(10P/1Q)/S.


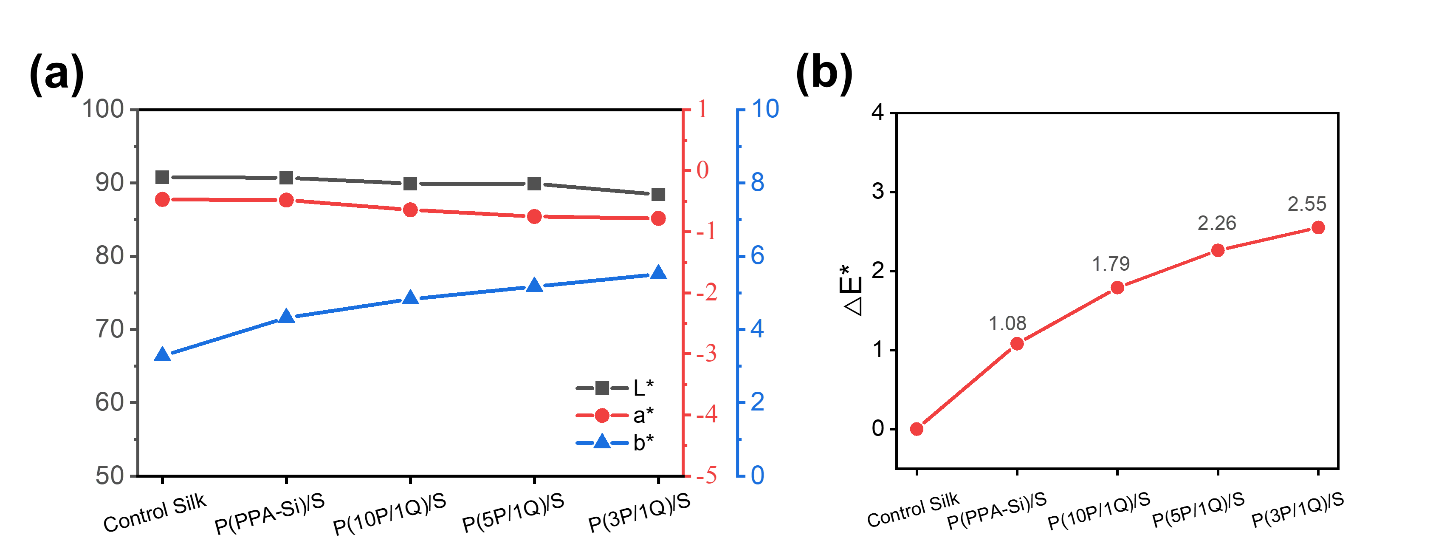


Fig. S7. Color difference between the control silk and P(PPA-Si/QAS)/S. Color difference values of control silk, P(PPA-Si)/S and P(P/Q)/S.


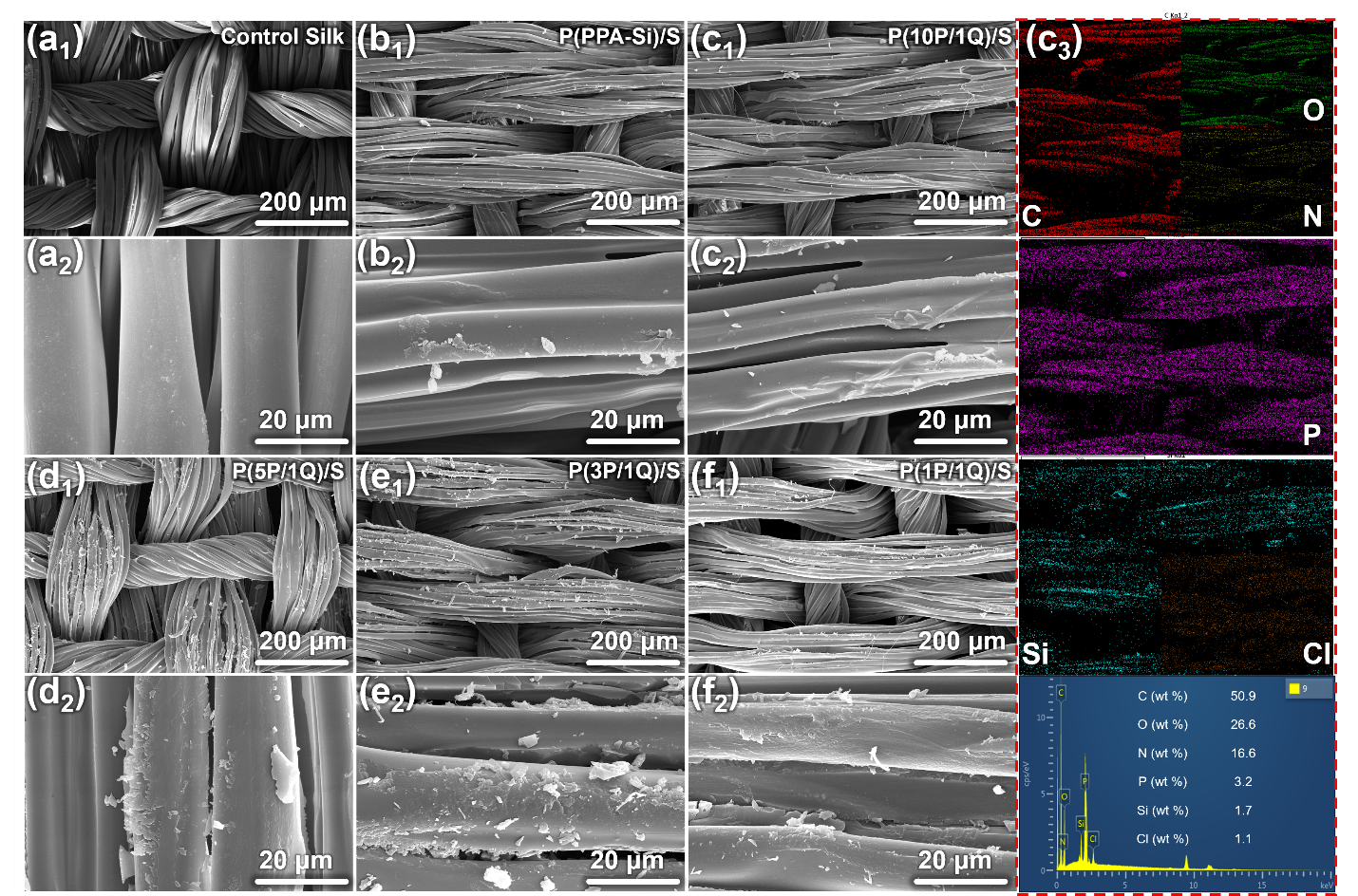


Fig. S8. Surface microstructure of control and surface treated silk. SEM micrographs of (a) control silk, (b) P(PPA-Si)/S, (c) P(10P/1Q)/S, (d) P(5P/1Q)/S, (e) P(3P/1Q)/S, and (f) P(1P/1Q)/S. (c_3_) EDS mapping of P(10P/1Q)/S.


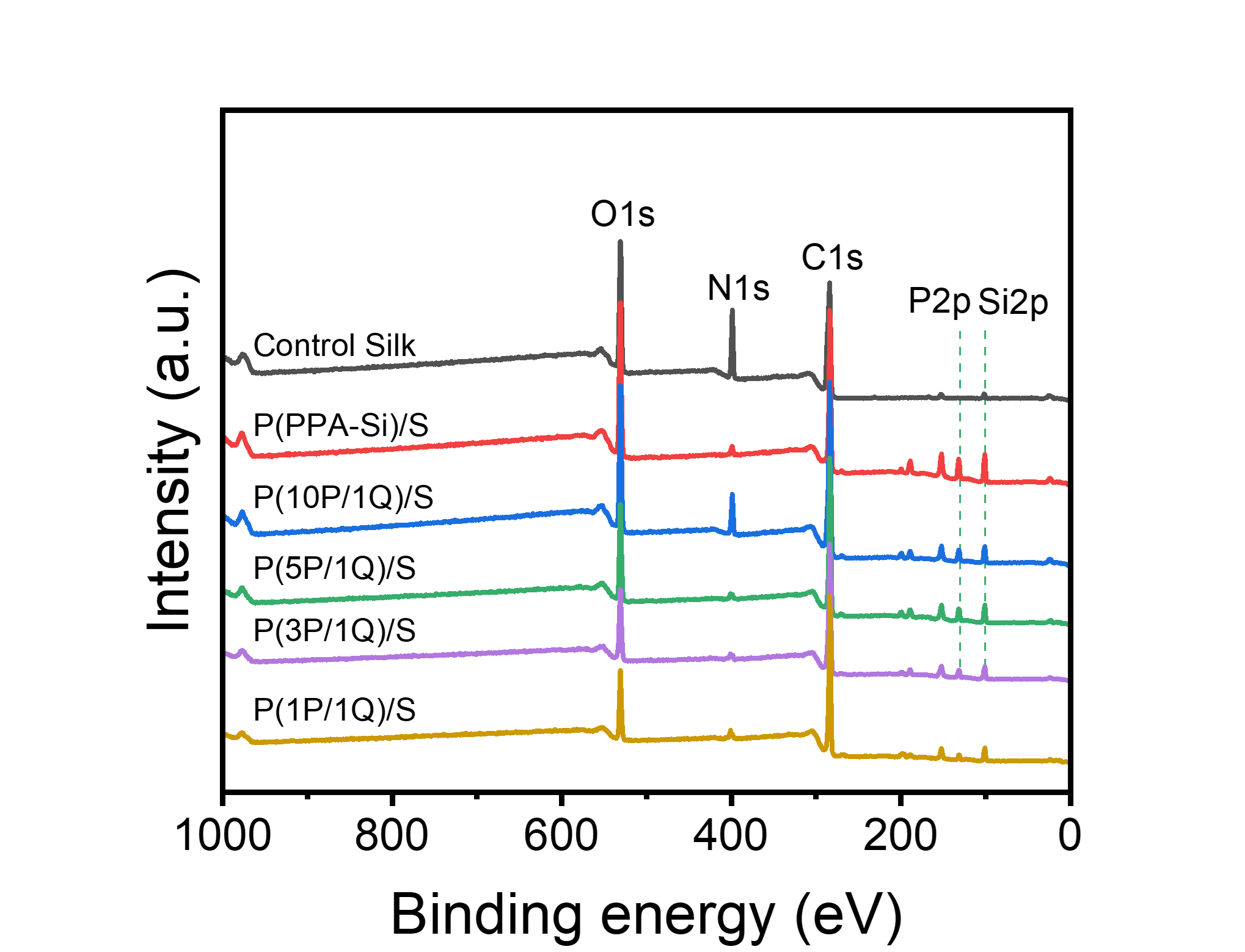


Fig. S9. XPS characterization of control and surface treated silk. Full survey XPS spectra of control silk, P(PPA-Si)/S and P(PPA-Si/QAS)/S.

Fig. S10. Flame retardancy of surface treated silk. (a-f) Digital photographs of the control silk, P(PPA-Si)/S and P(PPA-Si/QAS)/S after the vertical flame tests. (g) Digital photograph of the large-scale surface treated silk textile with a size of 110 cm × 60 cm (LP(10P/1Q)/S). (h) Digital photographs of the LP(10P/1Q)/S after vertical flame tests.


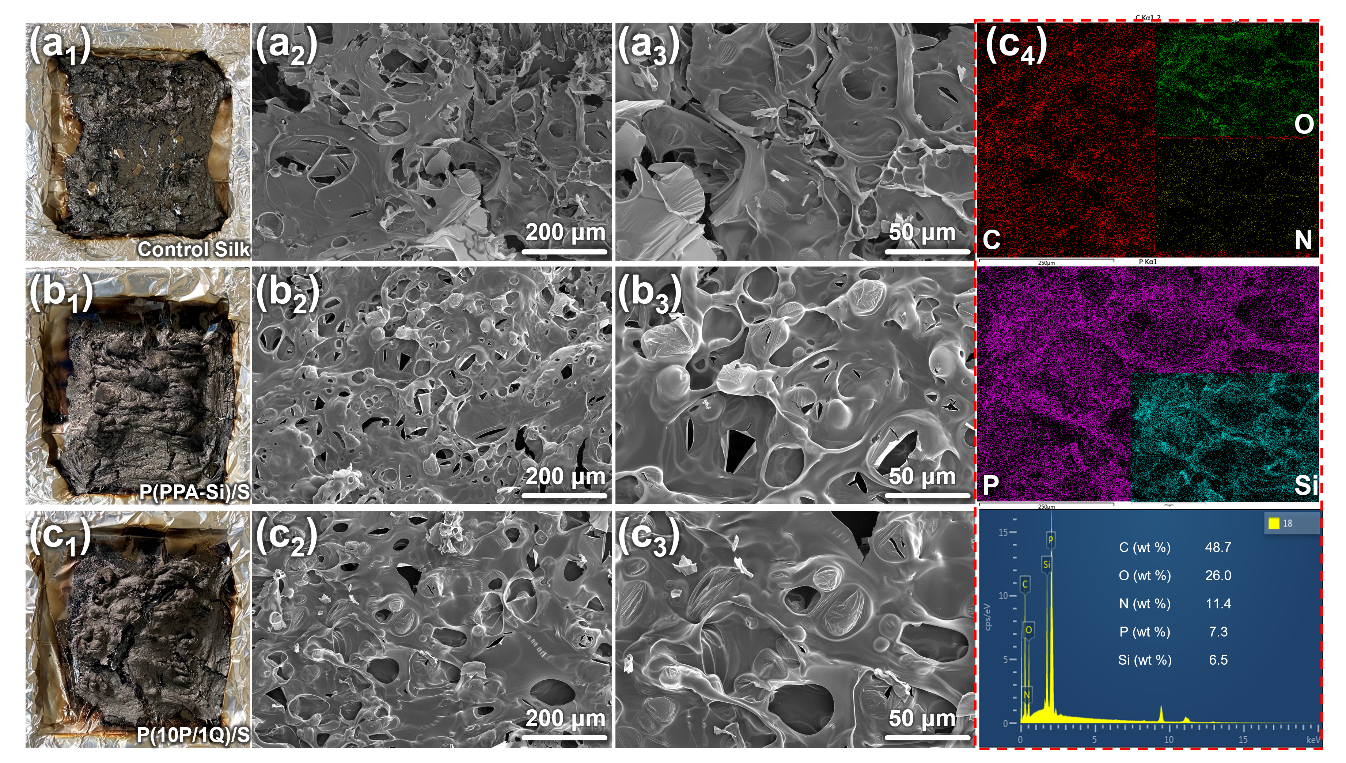


Fig. S11. Digital photographs and surface microstructure of control and surface treated silk after cone calorimeter testing. Digital photographs and SEM micrographs of the residue chars for (a) control silk, (b) P(PPA-Si)/S, and (c) P(10P/1Q)/S after cone calorimeter testing. (c_4_) EDS mapping of P(10P/1Q)/S after cone calorimeter testing.

**Fig. S11** displayed digital photographs, SEM images, and EDS mapping images of the control silk, P(PPA-Si)/S, and P(10P/1Q)/S after the CCT test. After combustion, a small amount of thin layer-like char residue remained in the control silk, and the microstructure consisted of disordered concave layers. In contrast, P(PPA-Si)/S and P(10P/1Q)/S exhibited a significantly larger char residue with an expansion structure, which may be attributed to the release of noncombustible volatile gas products during combustion. The microstructure indicated a noticeable increase in the thickness of the char layer and the formation of porous structures, which created a loosely packed porous char layer that blocked heat transfer and inhibited rapid degradation of the silk at high temperatures. EDS mapping revealed that the chemical composition of the char layer included P and Si, indicating the involvement of surface modification in the formation of the char layer and the condensation-domination mechanism.


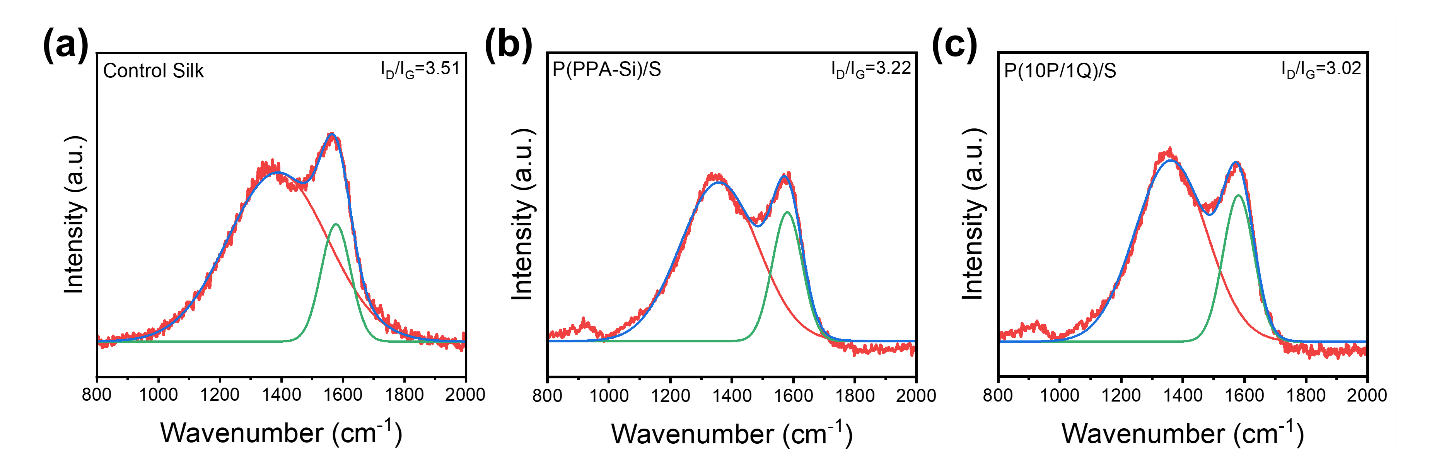


Fig. S12. Raman characterization of control and surface treated silk after cone calorimeter testing. Raman curves of the residue chars for (a) control silk, (b) P(PPA-Si)/S, and (c) P(10P/1Q)/S after cone calorimeter testing.

Raman spectral analysis was performed to determine the char composition and structure. In the Raman spectra, there were two peaks at 1362 cm^-1^ (D band) and 1586 cm^-1^ (G band), which represent disordered carbon and sp^2^-hybridized carbon atoms [1], respectively. The intensity ratio of D to G (I_D_/I_G_) was used to evaluate the degree of graphitization. Usually, a lower value of I_D_/I_G_ indicated better graphitization [2]. Herein, the I_D_/I_G_ value of the char residue for the control silk was 3.51. In contrast, after surface modification with P(PPA-Si) and P(10PPA-Si/1QAS), the corresponding char residues exhibited reduced I_D_/I_G_ values of 3.22 and 3.02, respectively. That was, the char residue formed after surface modification exhibited a higher degree of graphitization, implying enhanced thermal stability. This indicated that the modified silk textile can maintain its structural stability and continue to provide physical shielding at high temperatures.


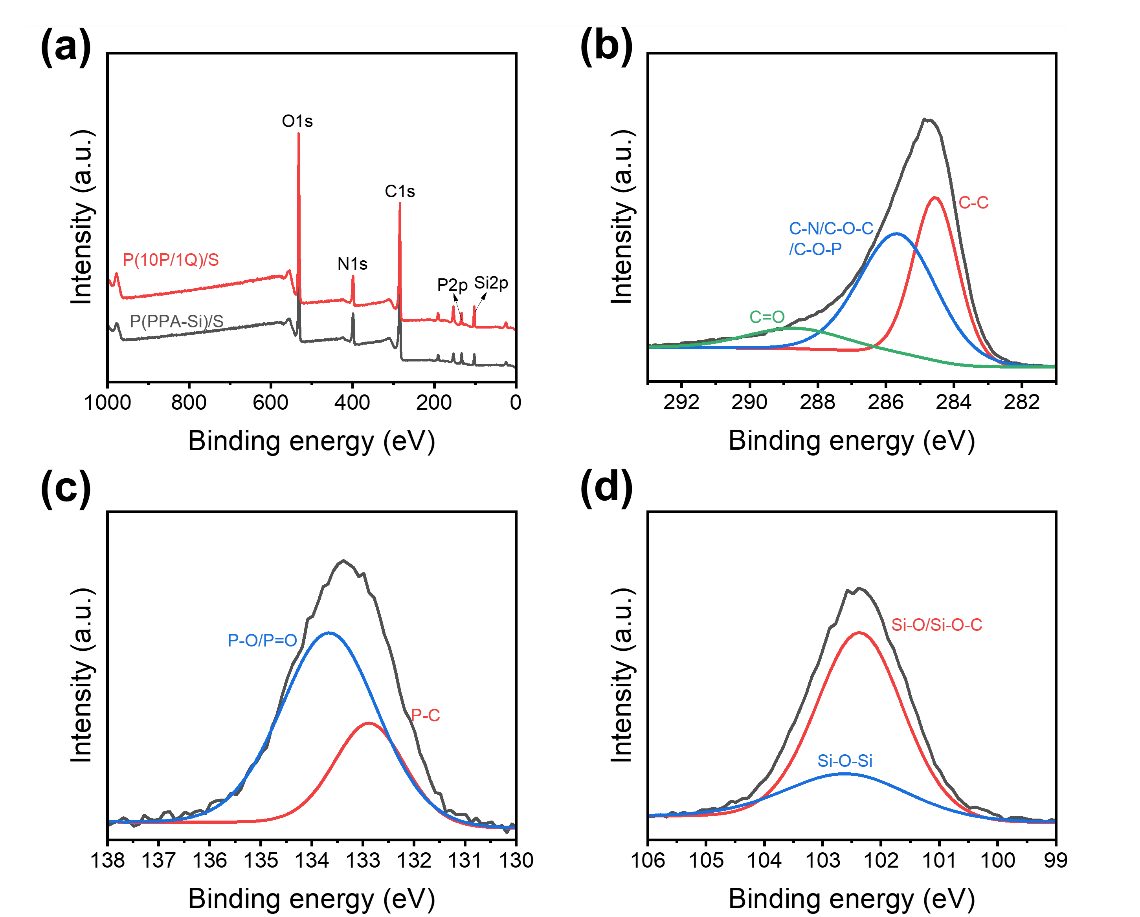


Fig. S13. XPS characterization of control and surface treated silk after cone calorimeter testing. (a) Full survey XPS spectra of P(PPA-Si)/S and P(10P/1Q)/S. (b-d) High-resolution C1s, P2p, and Si2p spectra of the residue chars of P(10P/1Q)/S after cone calorimeter testing.

X-ray photoelectron spectroscopy (XPS) was used to further investigate the elemental composition of the char residues after the CCT test. As shown in **Fig. S13**, the char residues of both P(PPA-Si)/S and P(10P/1Q)/S exhibited detectable P and Si. Spectral analysis revealed the presence of C-N/C-O-P (285.7 eV) peaks in the C1s spectrum, P-O/P=O (133.6 eV) and P-C (132.8 eV) peaks in the P2p spectrum, and Si-O-Si (102.7 eV) and Si-O/Si-O-C (102.3 eV) peaks in the Si2p spectrum [3, 4]. These results indicated that P(PPA-Si/QAS) not only participated in the formation of the carbon layer structure but also became embedded in the carbon layer through covalent bonding, significantly enhancing the stability of the carbon structure and resulting in a long-lasting protective effect.


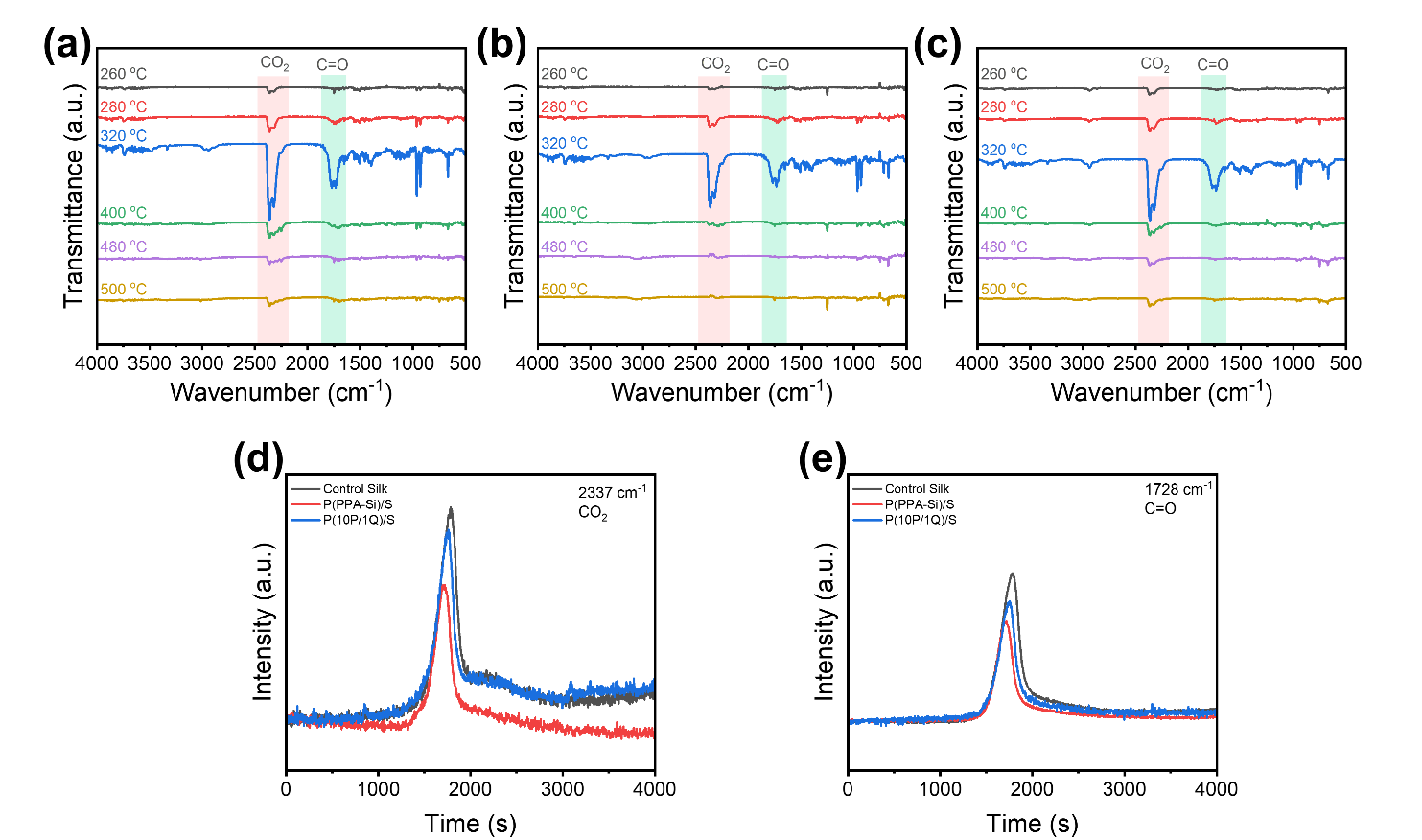


Fig. S14. TG-IR characterization of control and surface treated silk. FT-IR spectra of the released gases for (a) control silk, (b) P(PPA-Si)/S, and (c) P(10P/1Q)/S at selected temperatures. (d, e) Absorption intensities of the selected volatiles in the thermal degradation process of control and coated silk.

TG-IR spectra were obtained to further characterize the flame-retardant mechanism, and several FTIR spectra of the selected groups versus temperature were shown in **Fig. S14**. The main thermal decomposition gases released during the heating process of silk were the nonflammable gas CO_2_ and flammable carbonyl compounds. **Fig. S14(d, e)** showed that after modification with P(PPA-Si/QAS), the release of the nonflammable gas CO_2_ decreased, indicating a significant reduction in the degradation of the substrate at high temperatures [5, 6]. At the same time, the intensity of the release of flammable carbonyl compounds also decreased. P(PPA-Si/QAS) can trap more flammable gas products in condensed-phase char, effectively reducing the release of volatile flammable substances and interrupting the feedback of "fuel" to the flame, thereby suppressing the spread of fire [7].

In summary, according to the experimental results, P(PPA-Si/QAS) exhibited a condensed-phase flame-retardant mechanism for silk textiles. On one hand, surface modification promoted the formation of a thicker and more graphitized carbon layer during combustion, which could still provide physical shielding at high temperatures. On the other hand, the increased formation of the carbon layer meant that fewer volatile products were released into the air, reducing the fuel supply to the flame and slowing the rapid spread of the flame.


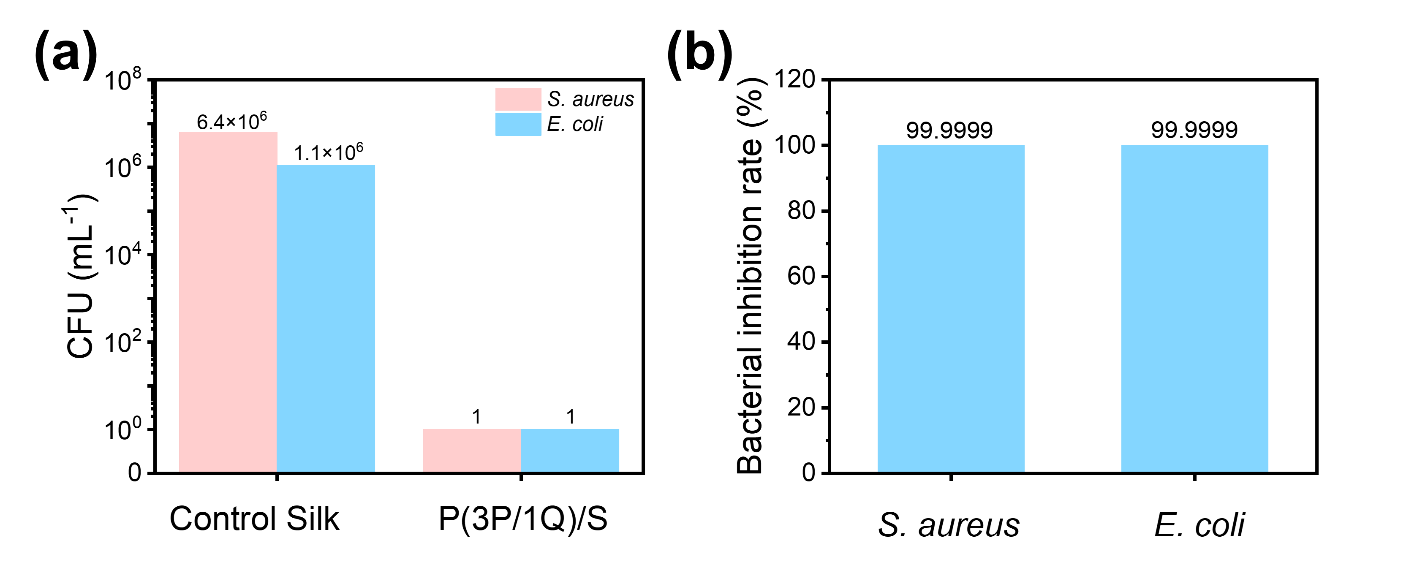


Fig. S15. Antibacterial property of multifunctional silk textiles. Antibacterial experimental results of control silk and P(3P/1Q)/S against *S. aureus* and *E. coli*, respectively.


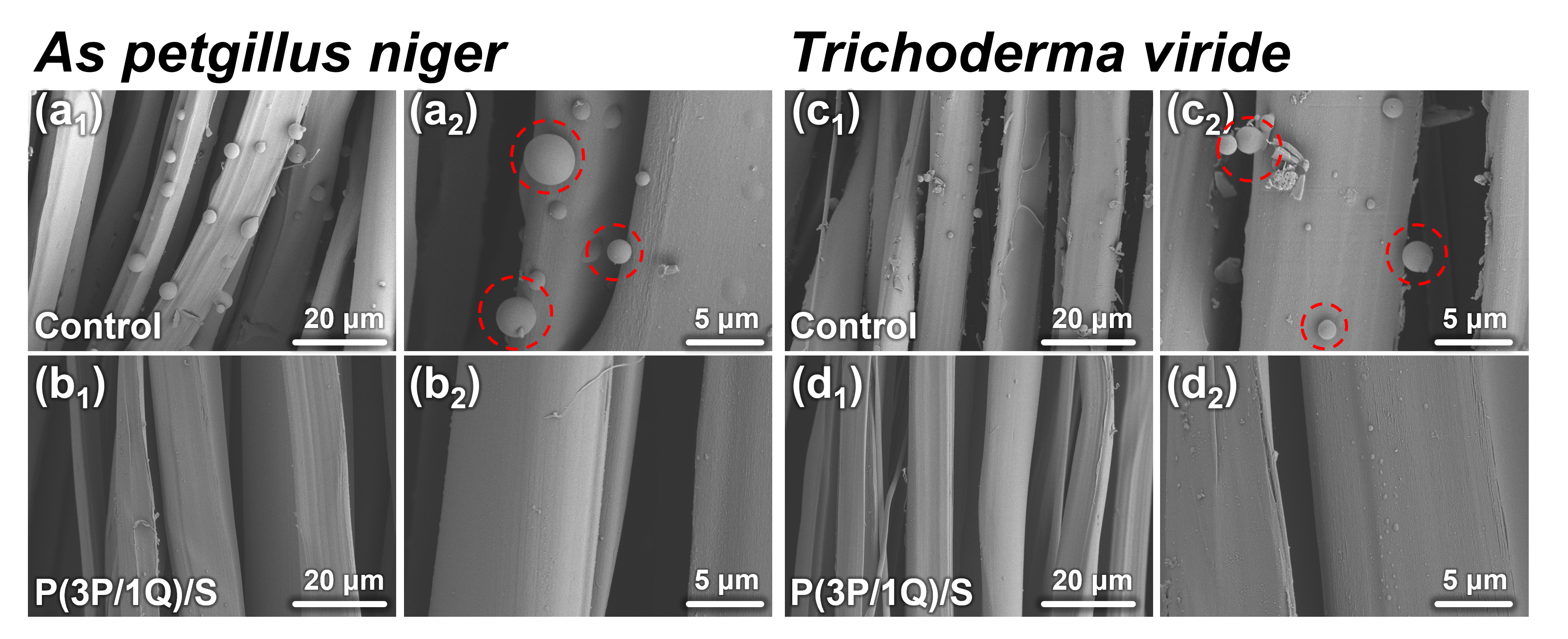


Fig. S16. Antimildew property of multifunctional silk textiles. SEM images of *As p. niger* and *T. viride* treated with (a, c) control silk and (b, d) P(3P/1Q)/S, respectively.


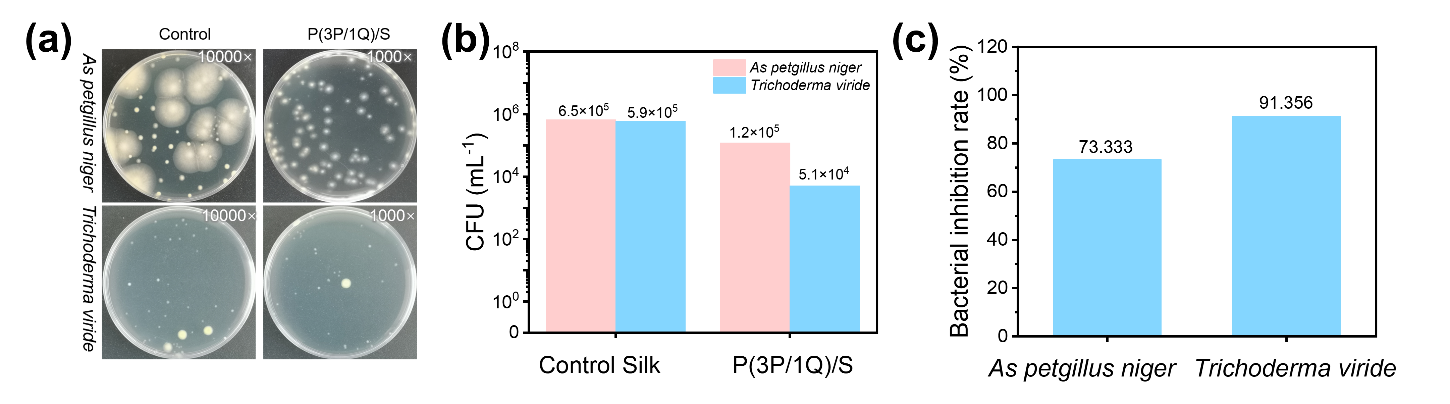


Fig. S17. Antimildew property of multifunctional silk textiles. Antimildew experimental results of control silk and P(3P/1Q)/S against *As p. niger* and *T. viride,* respectively.

To evaluate the antimicrobial performance, a plate counting measurement was conducted to investigate the antibacterial activity against *As p. niger* and *T. viride*. As shown in **Fig. S17**, after incubation, there were high-density bacterial colonies in culture media supplemented with control silk, while significantly reduced growth of the two bacterial strains treated with P(3P/1Q)/S was observed. The antibacterial activity of P(3P/1Q)/S reached 73.333% and 91.356% for *As p. niger* and *T. viride*, respectively, exhibiting effective antimicrobial effects.


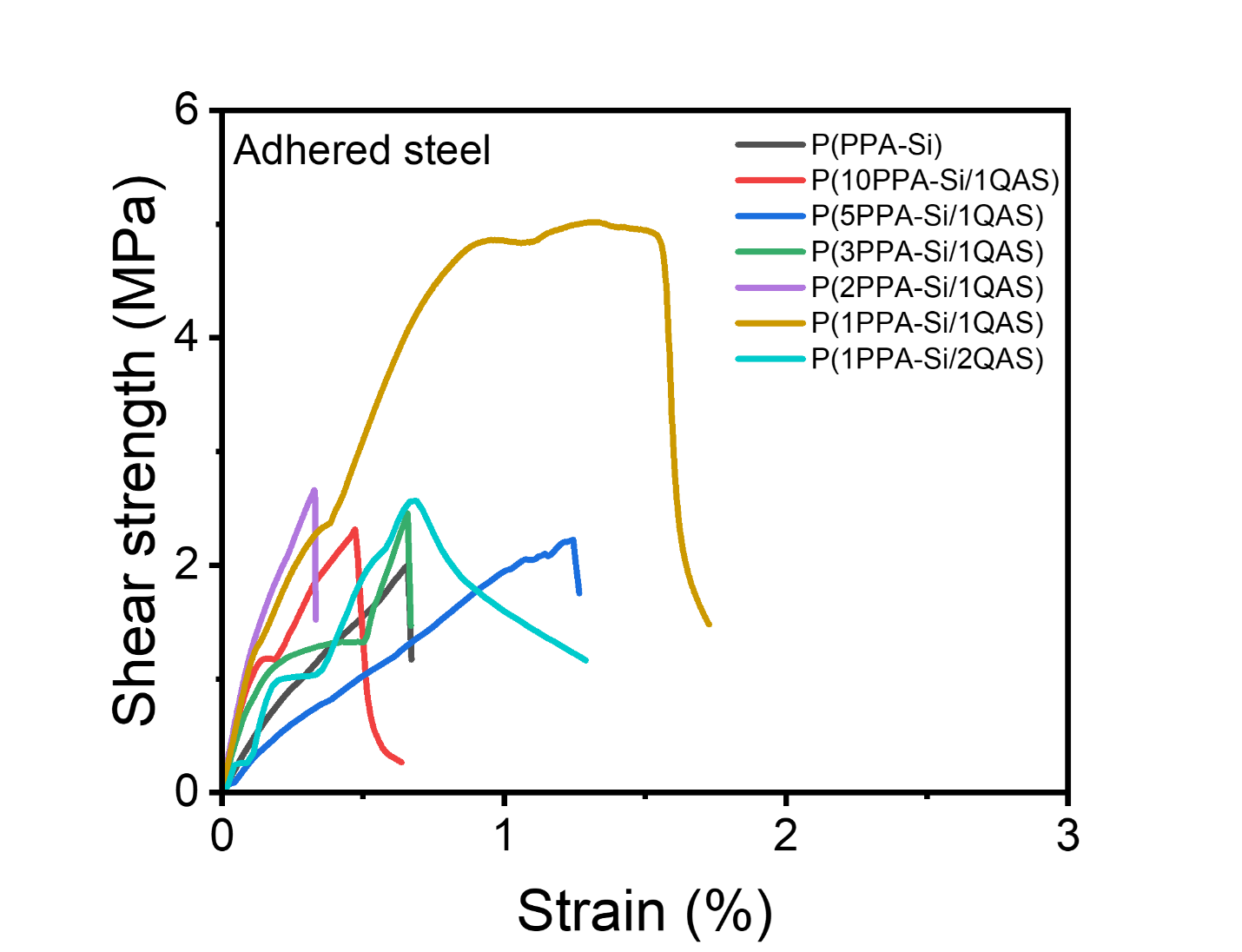


Fig. S18. Adhesion properties of P(PPA-Si) and P(PPA-Si/QAS) on steel substrates. Lap shear stress-strain curves of P(PPA-Si) and P(PPA-Si/QAS) on the steel substrate surface.


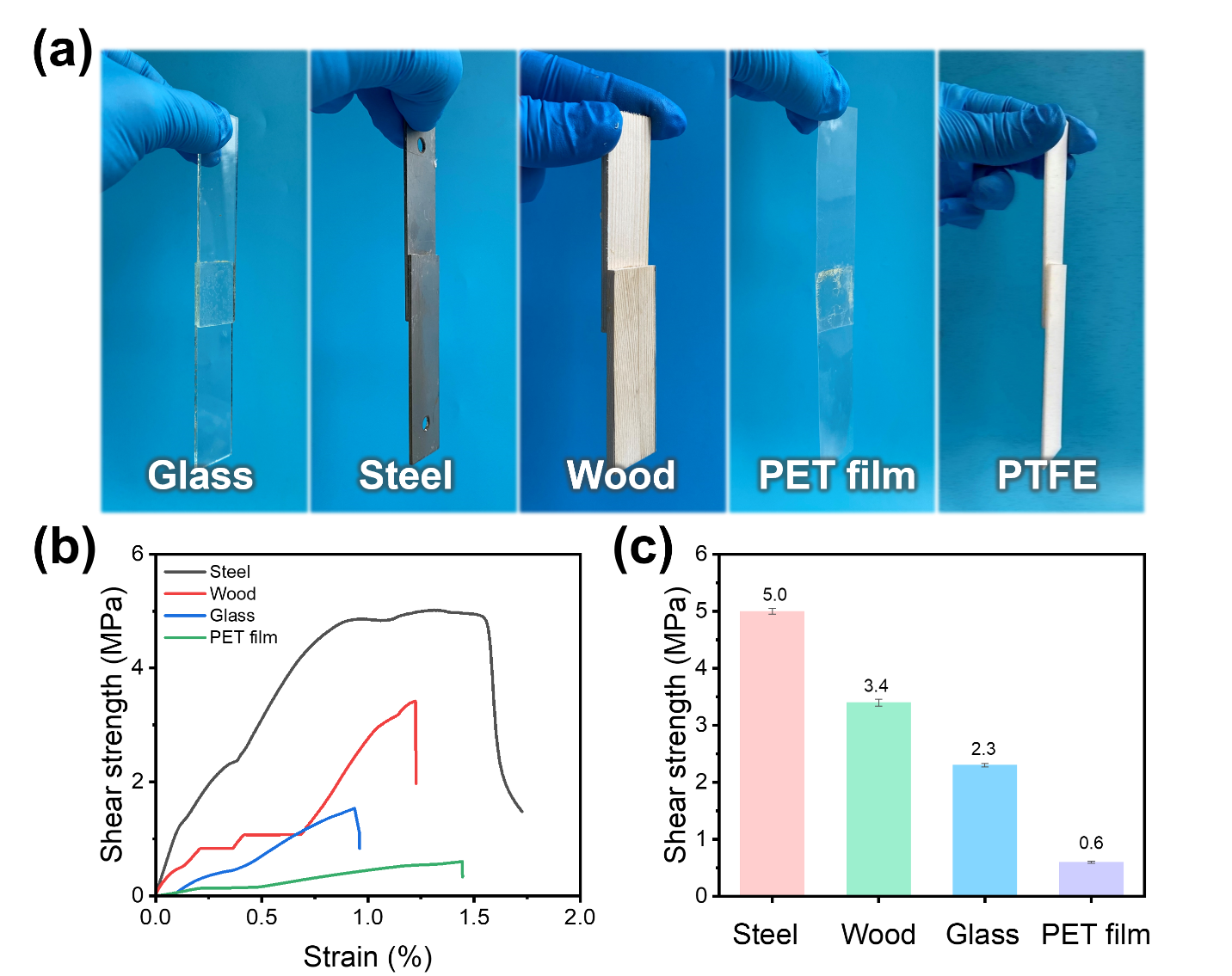


Fig. S19. Adhesion properties of P(1PPA-Si/1QAS) on different substrates. (a) Photographs of P(1PPA-Si/1QAS) adhered to various substrate surfaces (glass, steel, wood, PET film, PTFE). (b, c) Lap shear stress-strain curves and shear adhesion strength of P(1PPA-Si/1QAS) on various substrate surfaces (glass, steel, wood, PET film).


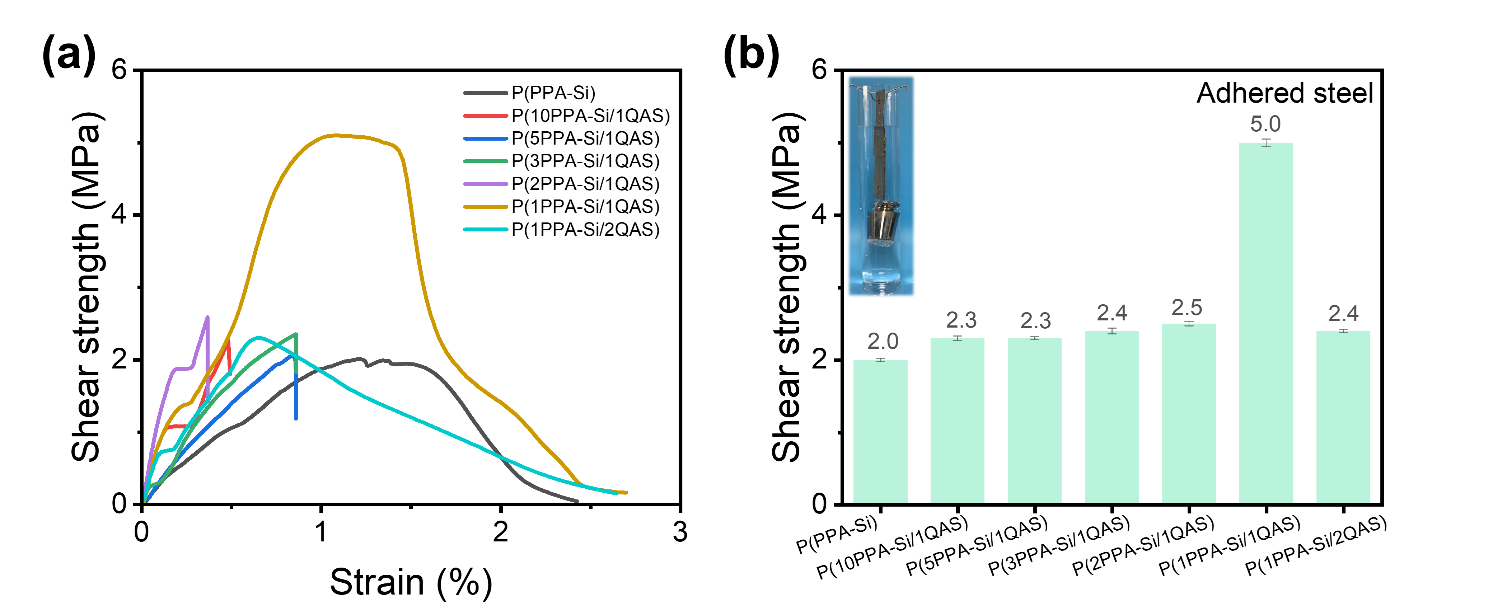


Fig. S20. Durable adhesion for P(PPA-Si) and P(PPA-Si/QAS) under water. Lap shear stress-strain curves and shear adhesion strength of P(PPA-Si) and P(PPA-Si/QAS) on the steel substrate surface after being soaked in water for 10 days.


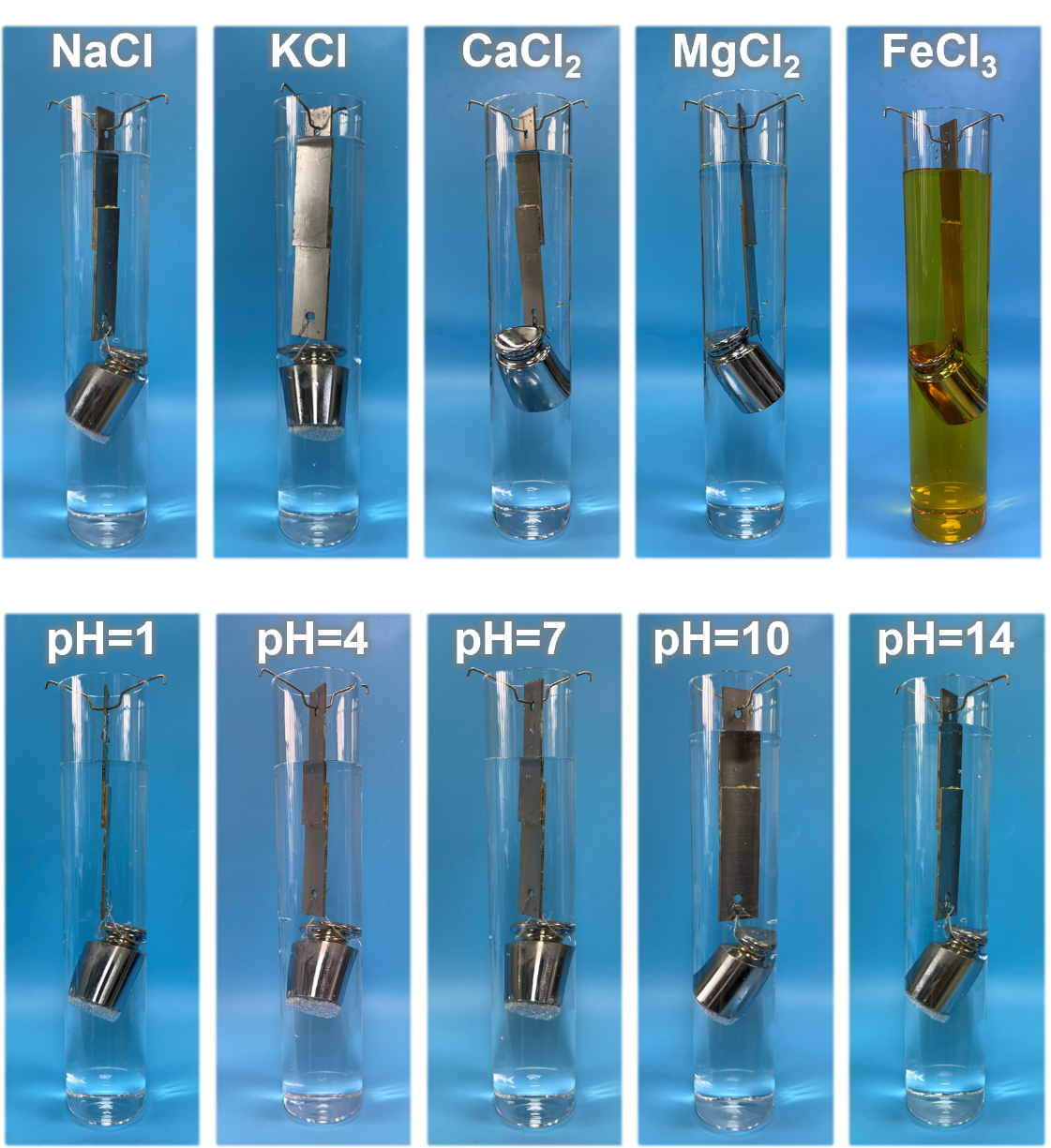


Fig. S21. Durable adhesion of P(10PPA-Si/1QAS) under various harsh environments. Photographs of P(10PPA-Si/1QAS) adhered on a steel substrate surface holding a 200 g weight loading after immersion in various salt/acid/alkaline solutions for 10 days.


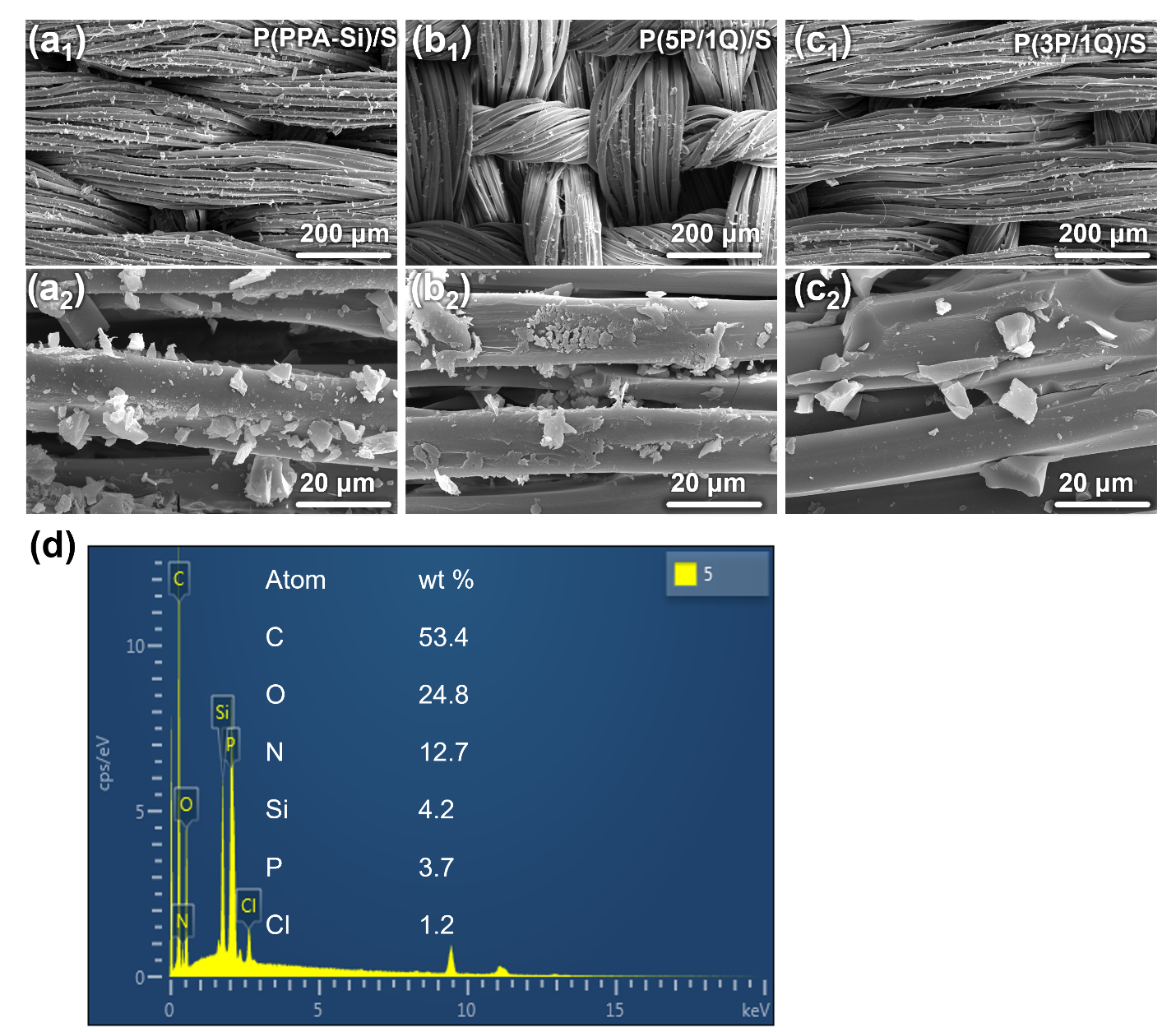


Fig. S22. Surface microstructure of P(PPA-Si)/S and P(PPA-Si/QAS)/S after immersion in water. SEM micrographs for (a) P(PPA-Si)/S, (b) P(5P/1Q)/S and (c) P(3P/1Q)/S after immersion in water for 10 days. (d) Element contents for P(10P/1Q)/S after immersion in water for 10 days.


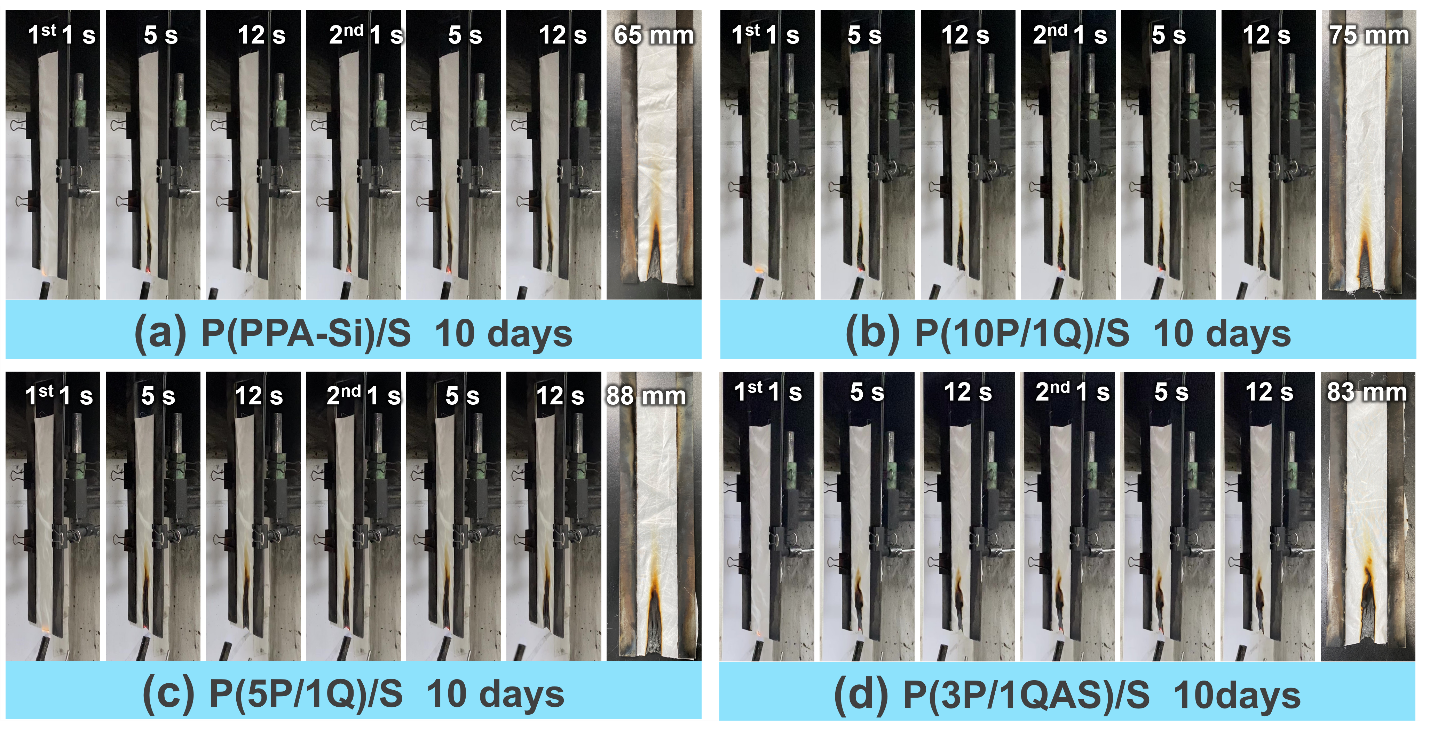


Fig. S23. Flame retardancy of P(PPA-Si)/S and P(PPA-Si/QAS)/S after immersion in water. Digital photographs after vertical flame tests for P(PPA-Si)/S and P(PPA-Si/QAS)/S after immersion in water for 10 days.


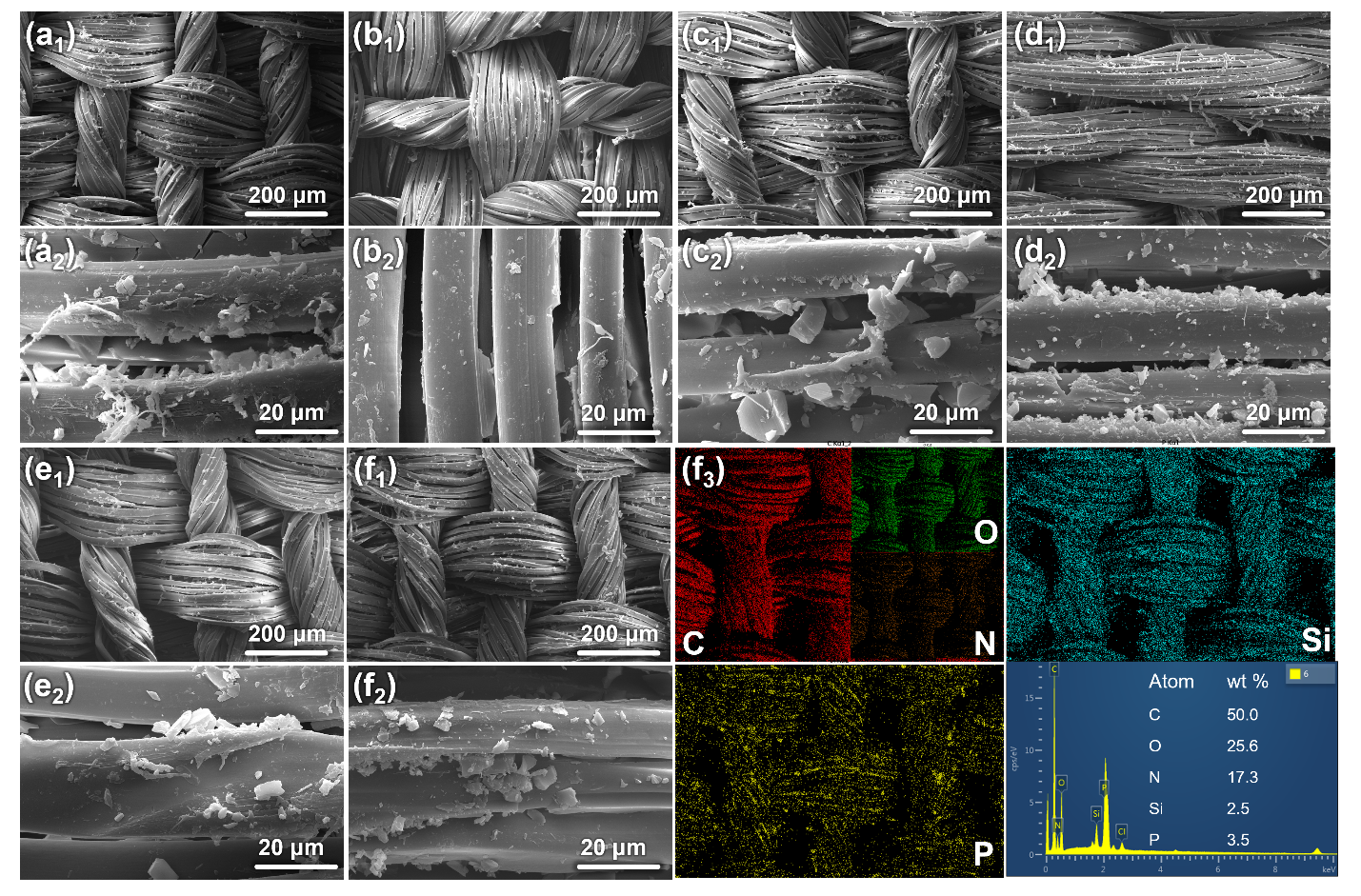


Fig. S24. Surface microstructure of P(10P/1Q)/S after immersion in different salt solutions. SEM micrographs for P(10P/1Q)/S after immersion in (a) NaCl, (b) KCl, (c) CaCl_2_, (d) MgCl_2_, (e) FeCl_3_ and (f) seawater solutions for 10 days. (f_3_) Element contents for P(10P/1Q)/S after immersion in seawater for 10 days.


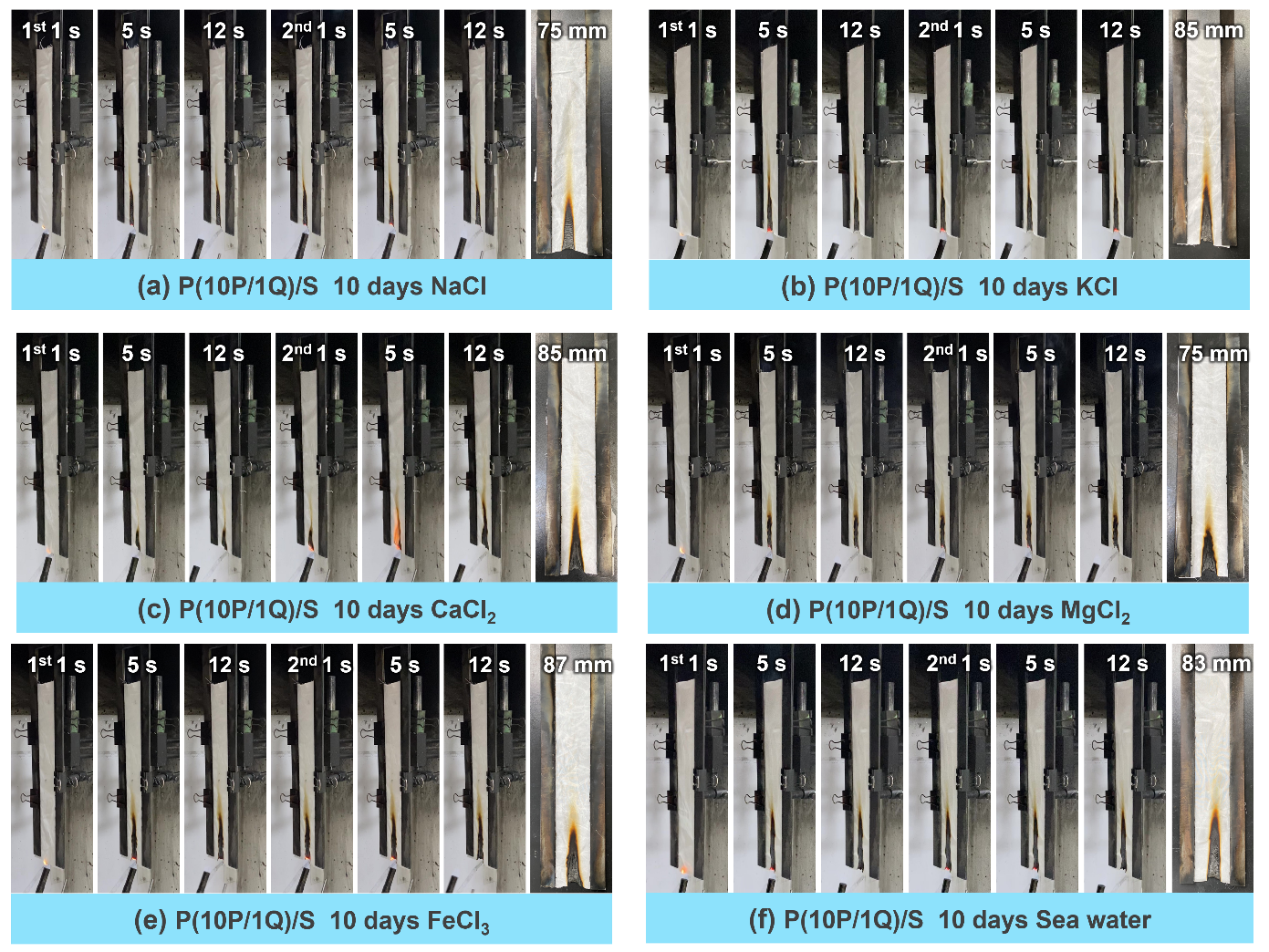


Fig. S25. Flame retardancy of P(10P/1Q)/S after immersion in different salt solutions. Digital photographs after vertical flame tests for P(10P/1Q)/S after immersion in different salt solutions (NaCl, KCl, CaCl_2_, MgCl_2_, FeCl_3_ and seawater) for 10 days.


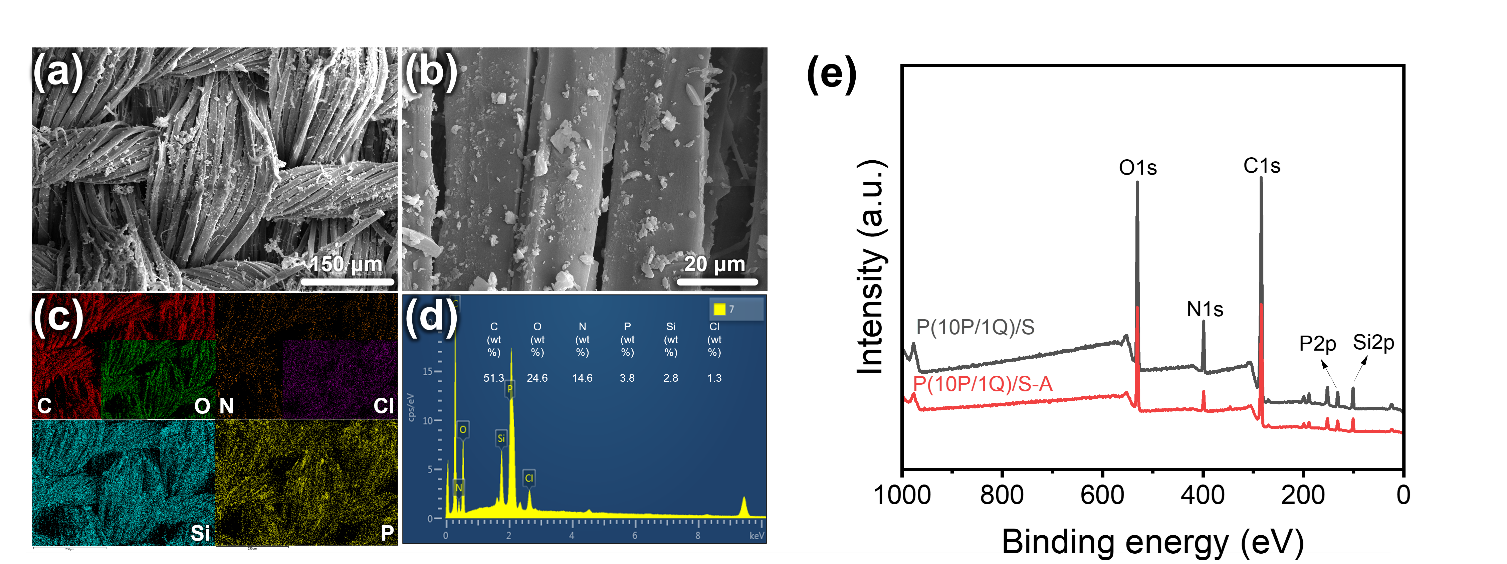


Fig. S26. Surface microstructure for P(10P/1Q)/S after the abrasion resistance test. (a-d) SEM micrographs and EDS mapping for P(10P/1Q)/S after the abrasion resistance test. (e) Full survey XPS spectra for P(10P/1Q)/S after the abrasion resistance test.


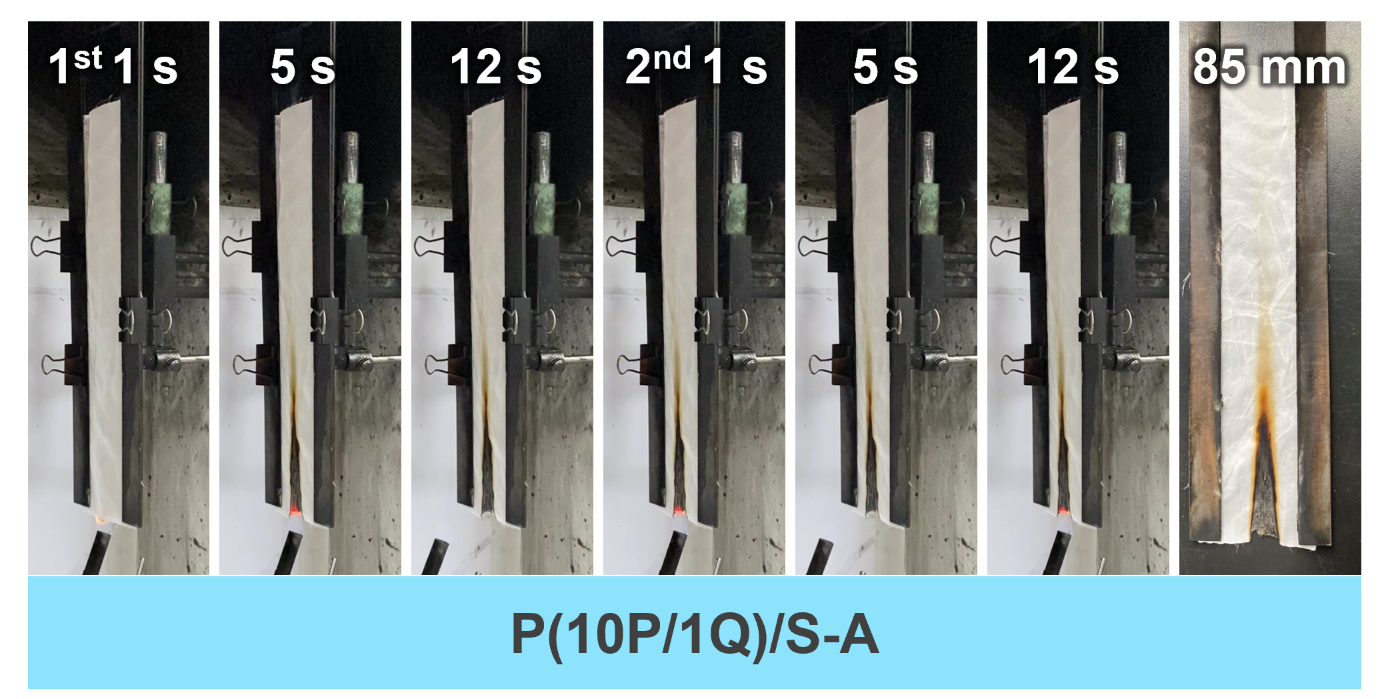


Fig. S27. Flame retardancy of P(10P/1Q)/S after the abrasion resistance test. Digital photographs after vertical flame tests for P(10P/1Q)/S after the abrasion resistance test.


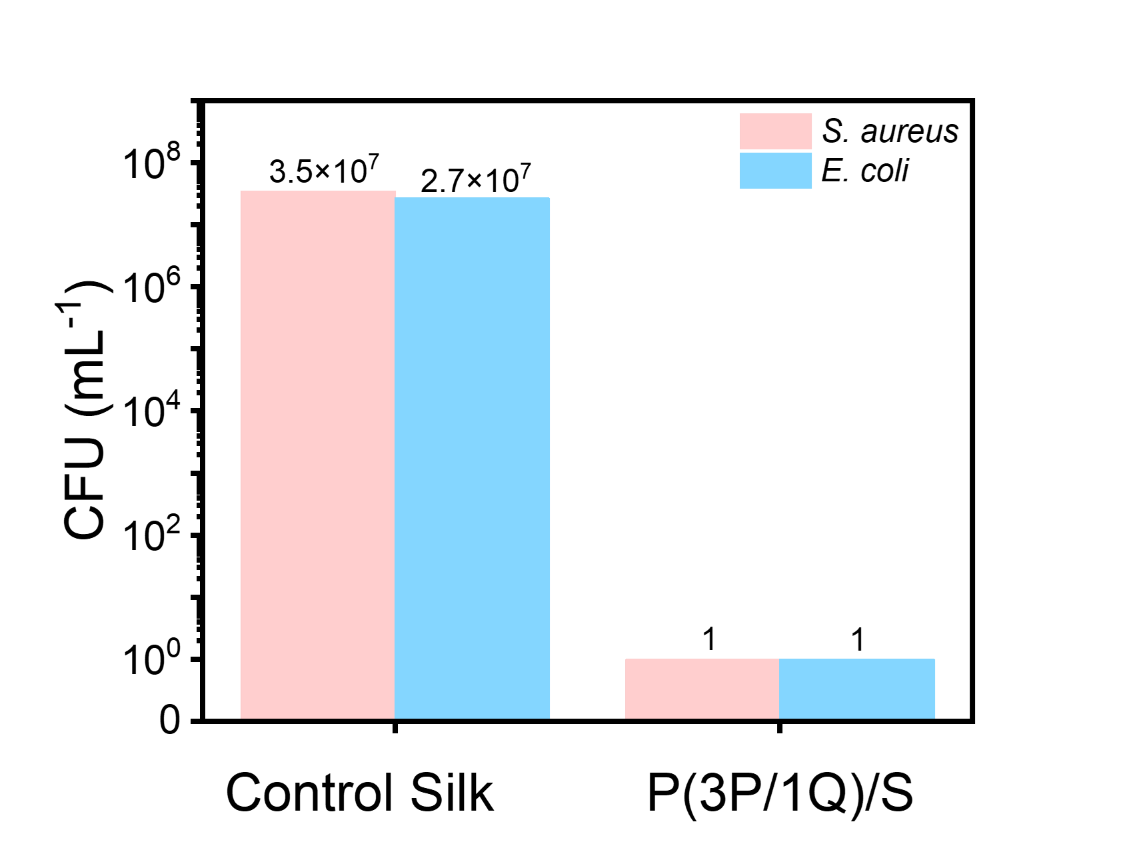


Fig. S28. Antibacterial properties of P(3P/1Q)/S after immersion in water. Antibacterial activity of control silk and P(3P/1Q)/S against *S. aureus* and *E. coli* after immersion in water for 10 days.


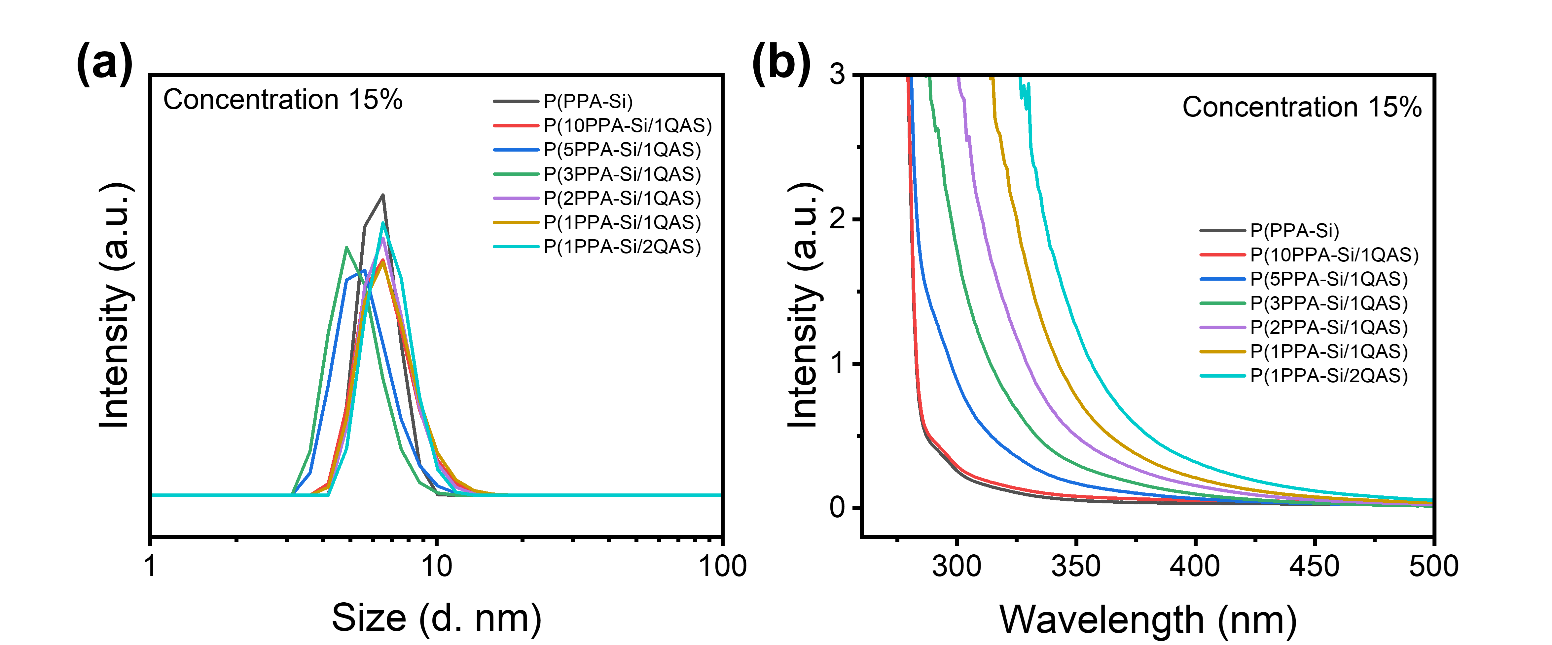


Fig. S29. Particle sizes and UV-vis spectra of different P(PPA-Si) and P(PPA-Si/QAS)/ethanol solutions. (a) Particle sizes and (b) UV-vis spectra for different P(PPA-Si) and P(PPA-Si/QAS)/ethanol solutions with different concentrations of 15%.


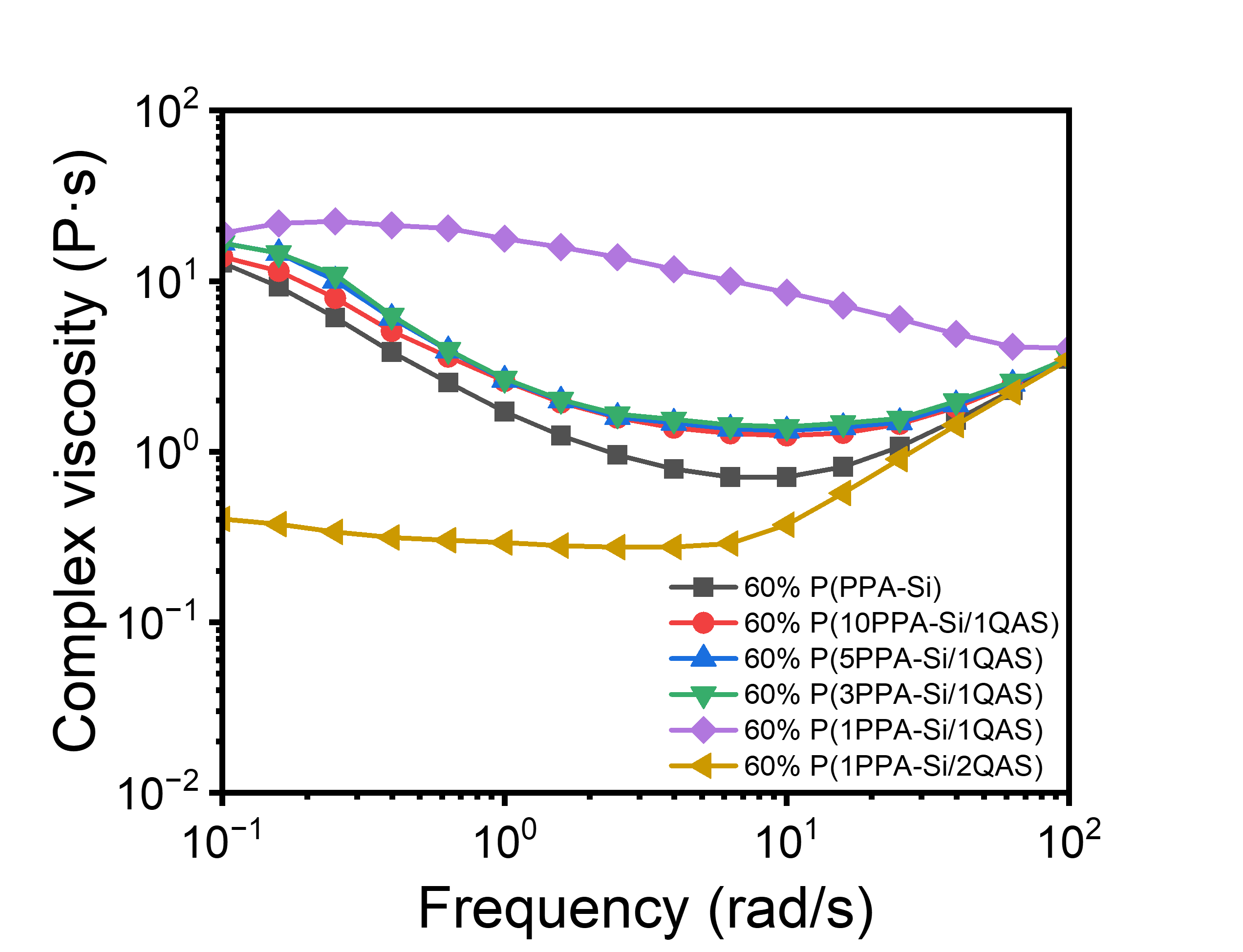


Fig. S30. Rheological behavior of different P(PPA-Si) and P(PPA-Si/QAS)/ethanol solutions. Frequency dependence of the complex viscosity for different P(PPA-Si) and P(PPA-Si/QAS)/ethanol solutions with different concentrations of 60%.


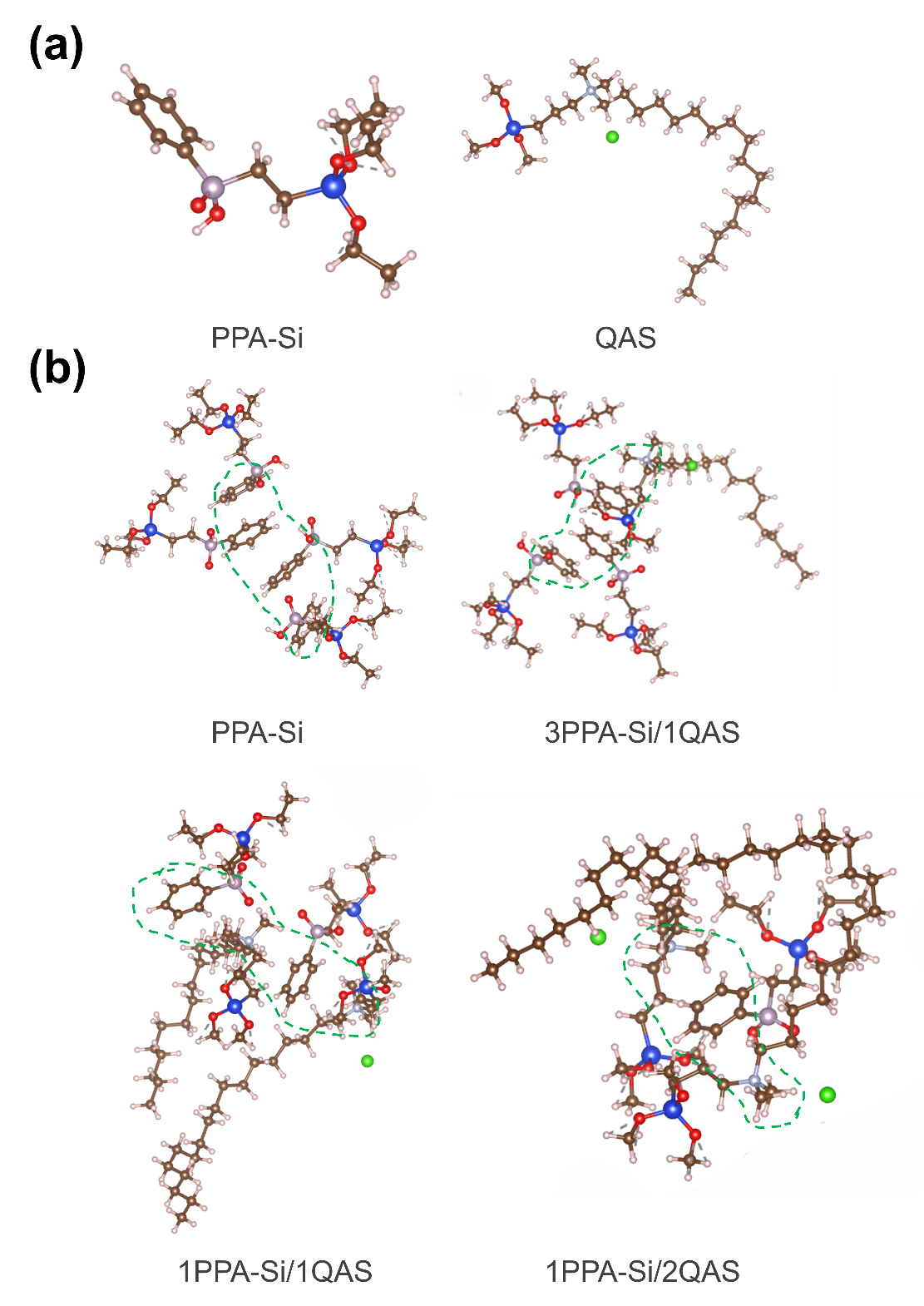


Fig. S31. DFT-optimized molecular models. (a) Molecular models of PPA-Si and QAS. (b) Cation-π interactions in DFT-optimized PPA-Si/QAS molecular models.


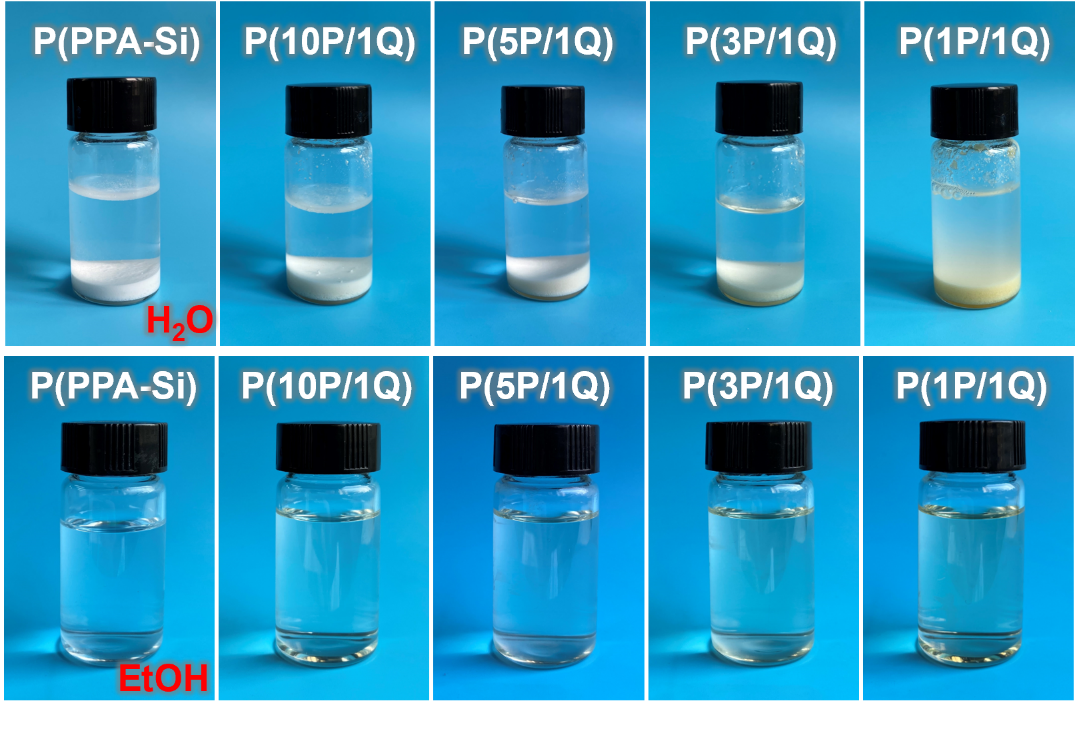


Fig. S32. Stability of P(PPA-Si) and P(PPA-Si/QAS) in water and ethanol. Digital photographs of P(PPA-Si) and P(PPA-Si/QAS) after being soaked in water and ethanol for 7 days.


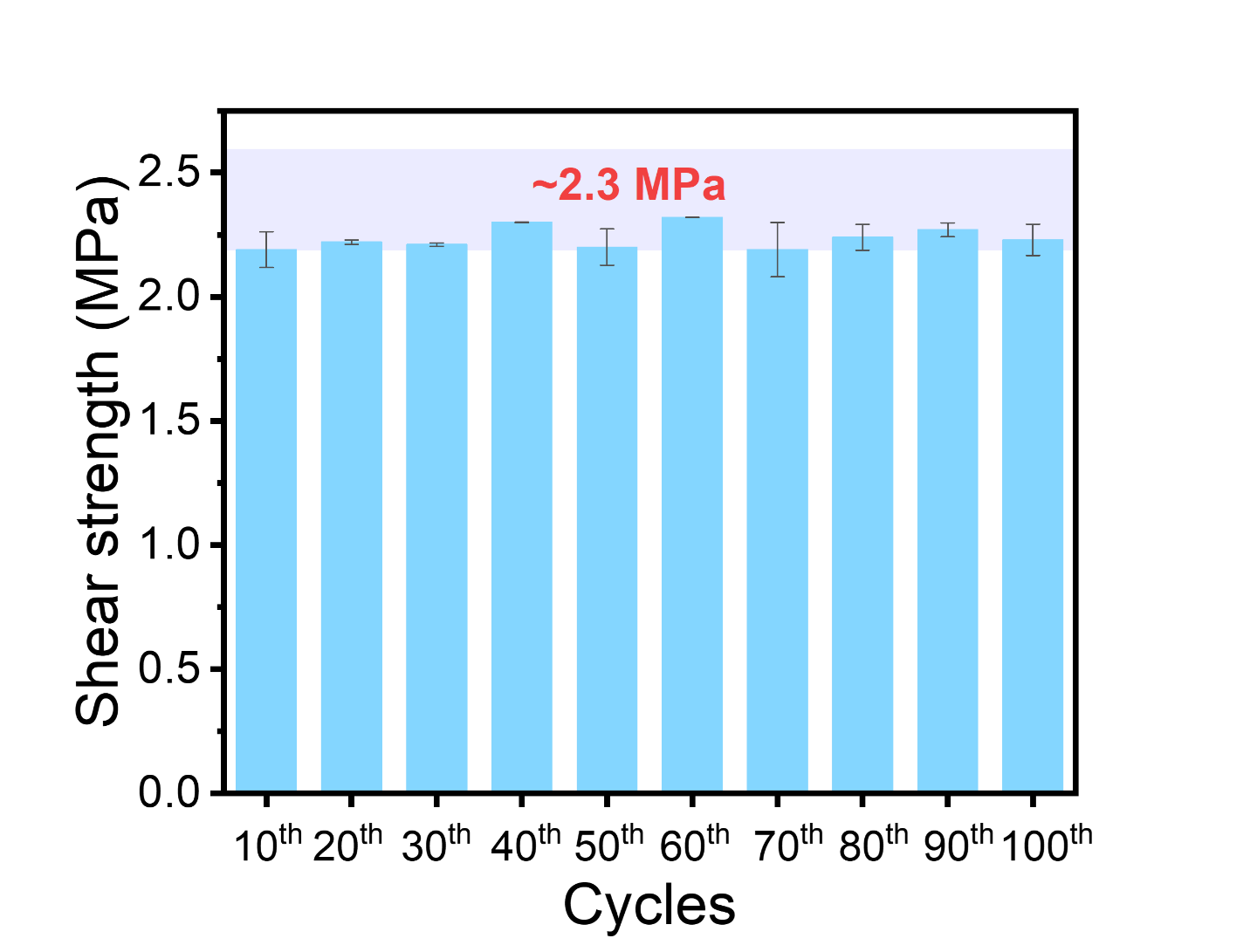


Fig. S33. Reversibility and reusability of P(10PPA-Si/QAS) adhesion. Shear adhesion strength of P(10PPA-Si/1QAS) after different numbers of reused adhesion cycles on steel surfaces.


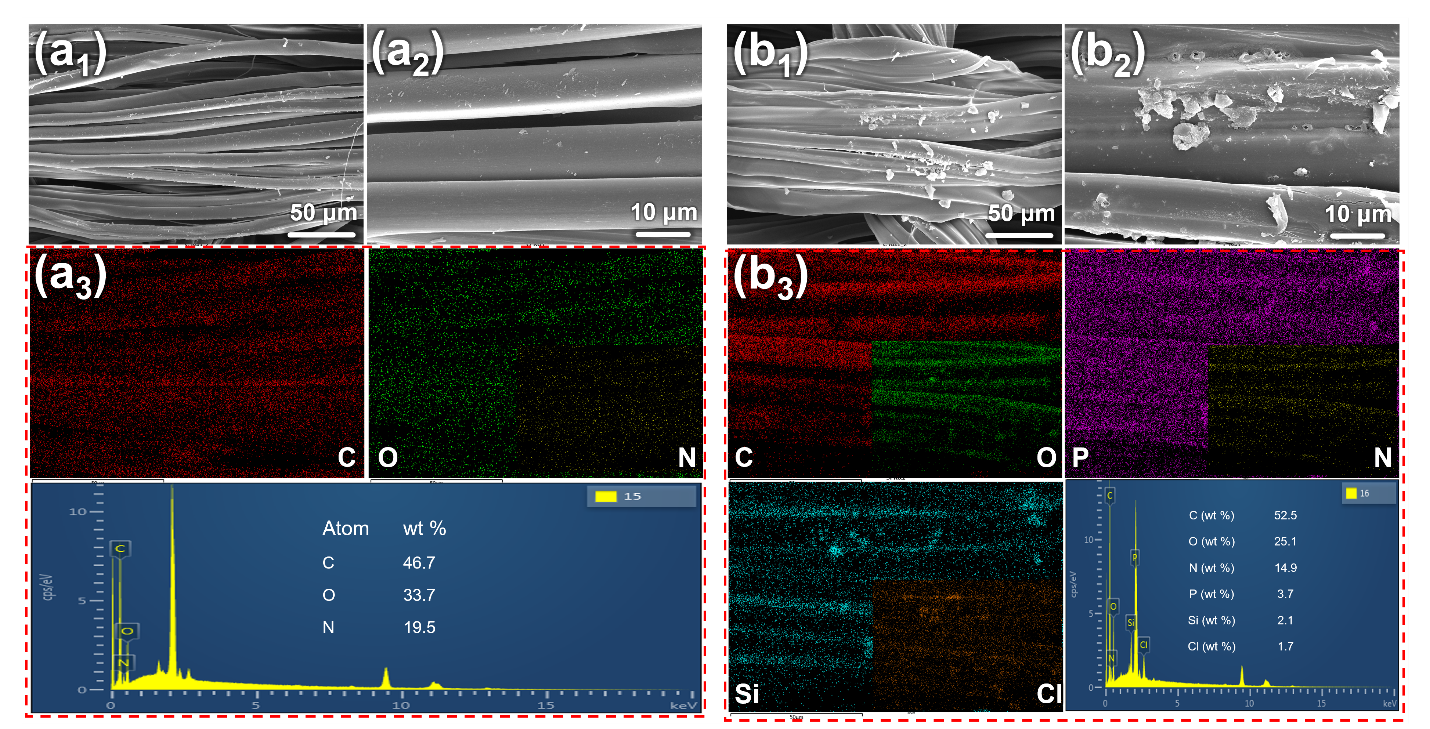


Fig. S34. Surface microstructure after the sustainable application of multifunctional silk textiles. (a) SEM micrographs and EDS mapping for P(10P/1Q)/S after soaking in excess ethanol. (b) SEM micrographs and EDS mapping for recovered P(3P/1Q)/S.


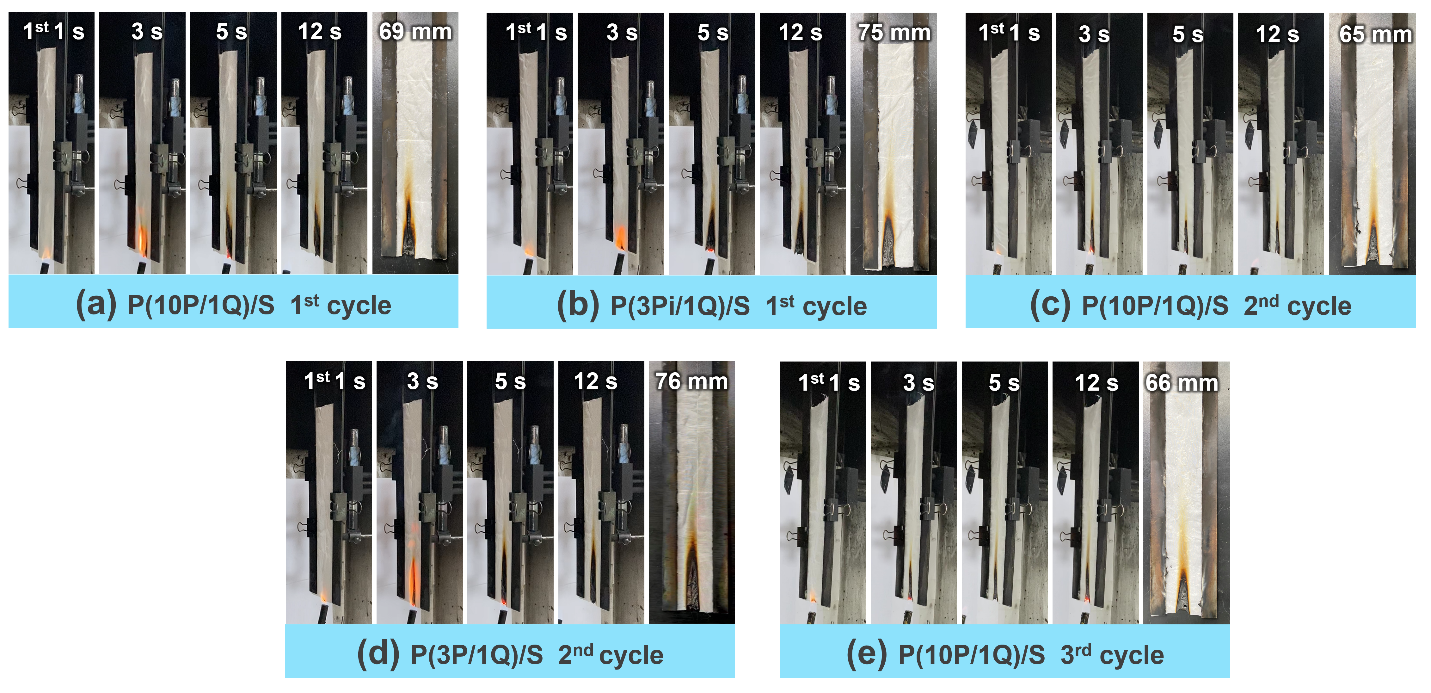


Fig. S35. Flame retardancy after sustainable application of multifunctional silk textiles. Digital photographs after the vertical flame tests after the sustainable and programmable application of treated silk.


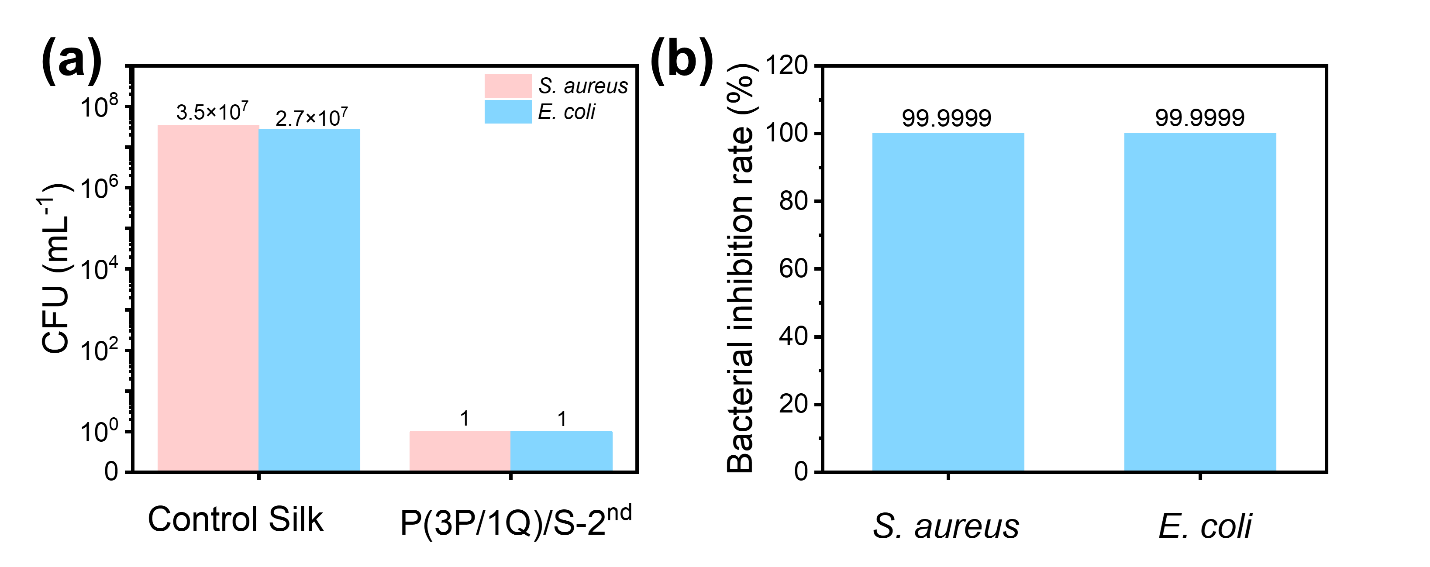


Fig. S36. Antibacterial property after sustainable application of multifunctional silk textiles. Antibacterial experimental results of control silk and P(3P/1Q)/S-2^nd^ against *S. aureus* and *E. coli*, respectively.


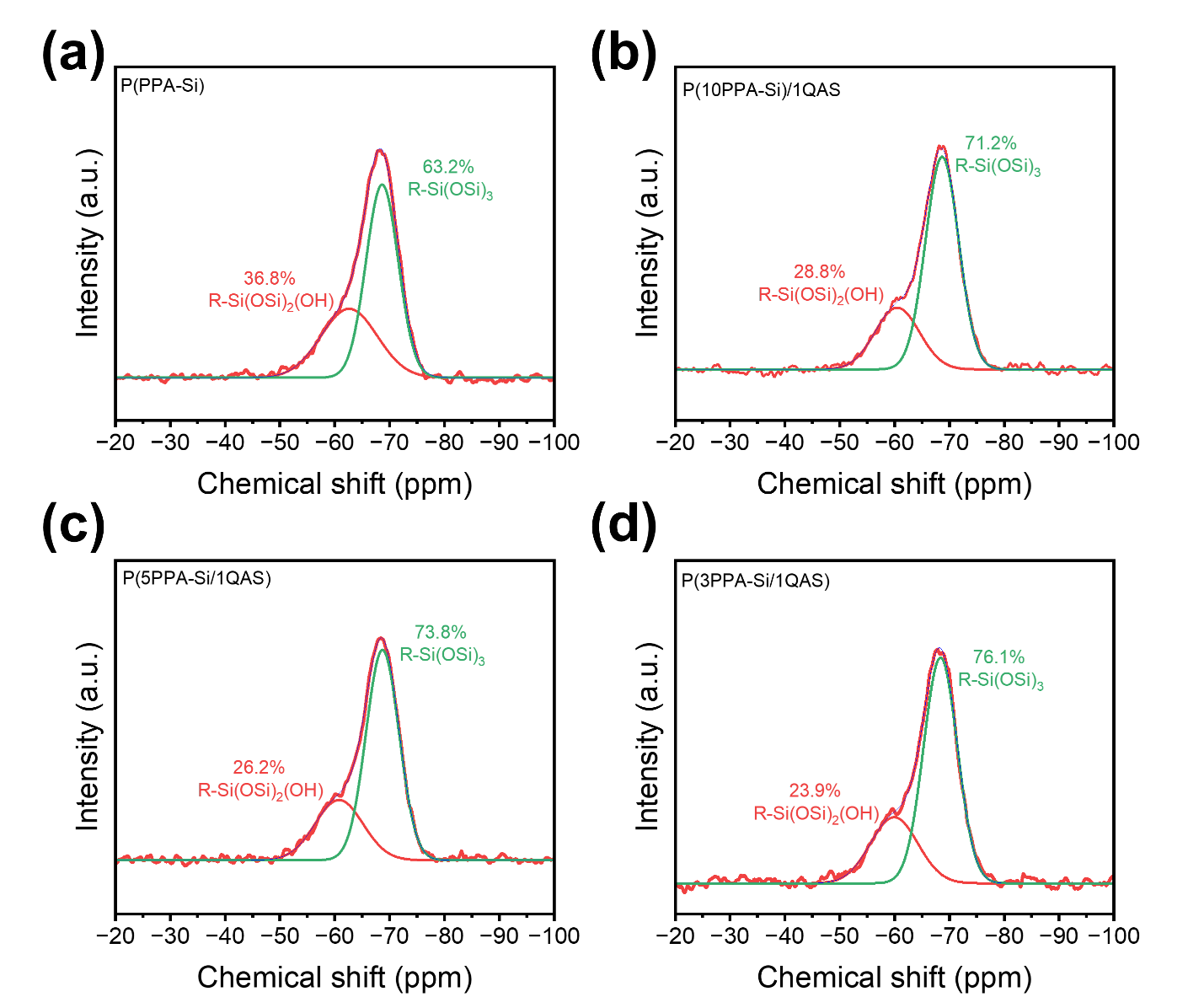


Fig. S37. Solid-state ^29^Si NMR characterization. ^29^Si magic angle spinning NMR spectra of P(PPA-Si) and P(PPA-Si/QAS).


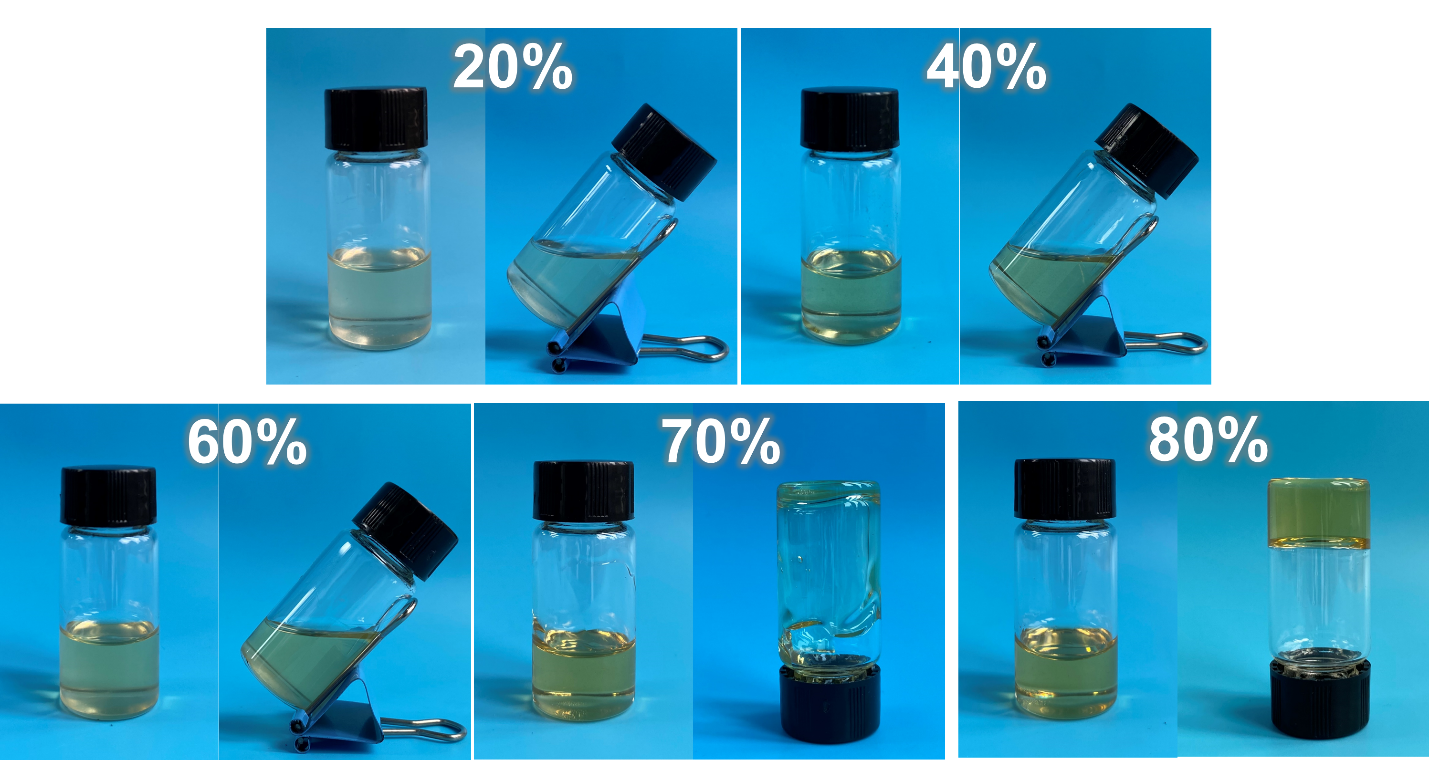


Fig. S38. Dissolution behavior and rheological behavior for different P(10PPA-Si/1QAS)/ethanol solutions with different concentrations (20%, 40%, 60%, 70% and 80%).


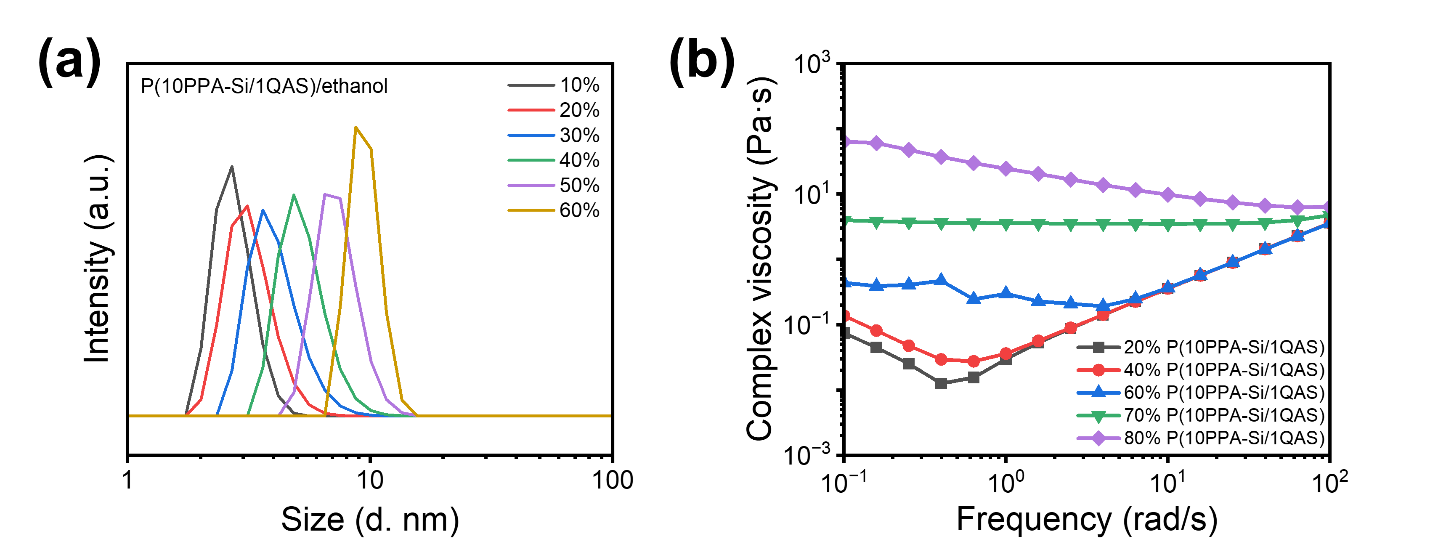


Fig. S39. Particle sizes and rheological behavior for different P(10PPA-Si/1QAS)/ethanol solutions. (a) Particle sizes of nanomicelles for different concentrations of P(10PPA-Si/1QAS)/ethanol solutions. (b) Frequency dependence of the complex viscosity for P(10PPA-Si/1QAS)/ethanol solutions with different concentrations (20%, 40%, 60%, 70% and 80%).

Table S1. Formulations of silk textiles with different P(PPA-Si/QAS) functionalization

| Sample | Molar ratio (PPA-Si : QAS) | Ethanol/H_2_O (V/V) | Concentration (%) | WG (%) |
| --- | --- | --- | --- | --- |
| P(PPA-Si)/S | - | 2/1 | 20 | 21.0 |
| P(10P/1Q)/S | 10:1 | 2/1 | 20 | 22.8 |
| P(5P/1Q)/S | 5:1 | 2/1 | 20 | 23.5 |
| P(3P/1Q)/S | 3:1 | 2/1 | 20 | 19.8 |
| P(1P/1Q)/S | 1:1 | 2/1 | 20 | 23.3 |

Table S2. Comparison of P(PPA-Si/QAS) with other reported durable surface modification treatments in terms of sustainability, durable adhesion, antimicrobial properties, flame retardancy, and transparency

| Samples | Sustainability | Durability | Antimicrobial | Flame retardancy | Transparency (%) | Refs |
| --- | --- | --- | --- | --- | --- | --- |
| PTL-pSBMA | Fully recyclable | Superhydrophobic | - | - | ~100% | [8] |
| PVA/  MMT | - | - | - | Flame retardant to PU foam | 80%-100% | [9] |
| F-POSS  /Ag/PEI | Self-healing (16 cycles) | Superhydrophobic | Antibacterial | - | - | [10] |
| F-POSS  /APP/PEI | Self-healing (10 cycles)19 | Superhydrophobic | - | Flame retardant to cotton | - | [11] |
| CPHA | - | Resistance to water (washing for 30 cycles) | Antibacterial | - | - | [12] |
| Cu-IT | - | Excellent resistance to water (washing for 50 cycles) | Antibacterial and antiviral | - | - | [13] |
| Ta/TE/Fe | - | Resistance to water salt/acid (washing for 50 cycles) | - | Flame retardant to cotton | - | [7] |
| PDMP | - | Resistance to water (washing for 50 cycles) | - | Flame retardant to cotton | ~80% | [14] |
| NG | - | Resistance to water (washing for 50 cycles) | Antibacterial | Flame retardant to cotton | - | [15] |
| CAA-SA-GS | - | Superhydrophobic (washing for 10 cycles) | Antibacterial | - | - | [16] |
| PEI/APP-TMC | - | Resistance to water (washing for 300 cycles) | - | Flame retardant to cotton | - | [17] |
| Our work | Fully recyclable | Excellent resistance to water, salt/acid/alkali solutions (immersion for 10 days) | Antibacterial (99.9999%) and antimildew | Flame retardant to silk | 98%-100% |  |

Table S3. Color difference between the control silk and P(PPA-Si/QAS)/S

| Sample | L* | △L* | a* | △a* | b* | △b* | △E* |
| --- | --- | --- | --- | --- | --- | --- | --- |
| Control silk | 90.79 | 0 | -0.47 | 0 | 3.28 | 0 | 0 |
| P(PPA-Si)/S | 90.70 | -0.25 | -0.48 | -0.07 | 4.32 | 1.05 | 1.08 |
| P(10P/1Q)/S | 89.92 | -0.87 | -0.64 | -0.17 | 4.83 | 1.55 | 1.79 |
| P(5P/1Q)/S | 89.90 | -1.09 | -0.75 | -0.28 | 5.17 | 1.89 | 2.26 |
| P(3P/1Q)/S | 88.42 | -2.36 | -0.78 | -0.31 | 5.51 | -0.84 | 2.55 |

Table S4. LOI values and VFT test results for the control silk and P(PPA-Si/QAS)/S

| Sample | WG (%) | LOI (%) | Vertical flame test | | | |
| --- | --- | --- | --- | --- | --- | --- |
|  |  |  | After-flame time (s) | After-glow time (s) | Char length (mm) | Pass or fail |
| Control Silk | - | 25.5±0.1 | 6 | 0 | Burn out | fail |
| P(PPA-Si)/S | 21.0 | 29.5±0.1 | 0 | 0 | 55±3 | pass |
| P(10/1Q)/S | 22.8 | 28.5±0.2 | 0 | 0 | 68±2 | pass |
| P(5/1Q)/S | 23.5 | 27.3±0.3 | 0 | 0 | 67±4 | pass |
| P(3/1Q)/S | 19.8 | 26.5±0.2 | 0 | 0 | 67±5 | pass |
| P(1/1Q)/S | 23.3 | - | 5 | 0 | 300 | fail |
| LP(10/1Q)/S | 20.1 | 28.4±0.1 | 0 | 0 | 80±3 | pass |

Table S5. Cone calorimetry results for the control silk and P(PPA-Si/QAS)/S

| Sample | TTI (s) | T_pHRR_ (s) | pHRR (kW/m^2^) | THR (MJ/m^2^) | Reduction in pHRR (%) | Residue (%) | FIGRA (kW/m^2^·s) |
| --- | --- | --- | --- | --- | --- | --- | --- |
| Control Silk | 5 | 15 | 121.9 | 1.1 | - | 22.2 | 8.1 |
| P(PPA-Si)/S | 5 | 15 | 80.9 | 0.9 | 33.6 | 32.6 | 5.3 |
| P(10/1Q)/S | 4 | 15 | 97.2 | 0.8 | 20.3 | 34.5 | 6.4 |
| P(5/1Q)/S | 4 | 15 | 103.2 | 1.0 | 15.3 | 31.7 | 6.8 |
| P(3/1Q)/S | 5 | 15 | 101.6 | 1.0 | 16.6 | 29.5 | 6.7 |

Table S6. Antimildew experimental results for P(3P/1Q)/S

| Model mildew | The coverage of model mildew on the sample surface | Antimildew rate |
| --- | --- | --- |
| *As p. niger* | ＜60% | 3 |
| *T. viride* | ＜10% | 1 |

Table S7. VFT test results for P(PPA-Si)/S and P(PPA-Si/QAS)/S after immersion in water for 10 days

| Sample | Weight remaining rate (%) | Vertical flame test | | | |
| --- | --- | --- | --- | --- | --- |
|  |  | After-flame time (s) | After-glow time (s) | Char length (mm) | Pass or fail |
| P(PPA-Si)/S | 95.7 | 0 | 0 | 65±2 | pass |
| P(10P/1Q)/S | 95.4 | 0 | 0 | 75±3 | pass |
| P(5P/1Q)/S | 94.6 | 0 | 0 | 88±4 | pass |
| P(3P/1Q)/S | 96.6 | 0 | 0 | 83±3 | pass |

Table S8. VFT test results for P(10P/1Q)/S after immersion in different salt solutions for 10 days

| Sample | Weight remaining rate (%) | Vertical flame test | | | |
| --- | --- | --- | --- | --- | --- |
|  |  | After-flame time (s) | After-glow time (s) | Char length (mm) | Pass or fail |
| NaCl | 95.3 | 0 | 0 | 75±3 | Pass |
| KCl | 95.8 | 0 | 0 | 85±2 | Pass |
| CaCl_2_ | 94.7 | 0 | 0 | 85±3 | Pass |
| MgCl_2_ | 96.0 | 0 | 0 | 75±2 | Pass |
| FeCl_3_ | 95.1 | 0 | 0 | 87±1 | Pass |
| Sea water | 94.3 | 0 | 0 | 83±4 | Pass |

Table S9. VFT test results for P(10P/1Q)/S against the abrasion resistance test for 50 cycles

| Sample | Weight remaining rate (%) | Vertical flame test | | | |
| --- | --- | --- | --- | --- | --- |
|  |  | After-flame time (s) | After-glow time (s) | Char length (mm) | Pass or fail |
| P(10P/1Q)/S | - | 0 | 0 | 68±2 | Pass |
| P(10P/1Q)/S-A | 97.5 | 0 | 0 | 85±3 | Pass |

Table S10. LOI values and VFT test results for each recoated P(PPA-Si/QAS)/S

| Sample | Recycle rate (%) | Weight gain (%) | LOI (%) | Vertical flame test | | | |
| --- | --- | --- | --- | --- | --- | --- | --- |
|  |  |  |  | After-flame time (s) | After-glow time (s) | Char length (mm) | Pass or fail |
| P(10P/1Q)/S 1^st^ cycle | 100 | 20.5 | 28.4±0.1 | 0 | 0 | 69±3 | Pass |
| P(3P/1Q)/S 1^st^ cycle | 100 | 21.8 | 26.6±0.2 | 0 | 0 | 75±2 | Pass |
| P(10P/1Q)/S 2^nd^ cycle | 100 | 21.4 | 28.3±0.2 | 0 | 0 | 65±3 | Pass |
| P(3P/1Q)/S 2^nd^ cycle | 100 | 22.0 | 26.5±0.1 | 0 | 0 | 76±2 | Pass |
| P(10P/1Q)/S 3^rd^ cycle | 100 | 21.0 | 28.5±0.2 | 0 | 0 | 66±4 | Pass |

Movie S1.

Combustion performance of the control silk.

Movie S2.

Combustion performance of the P(PPA-Si)/S.

Movie S3.

Combustion performance of the P(10P/1Q)/S.

Movie S4.

Combustion performance of the P(5P/1Q)/S.

Movie S5.

Combustion performance of the P(3P/1Q)/S.

**References**

1. A. Sadezky, H. Muckenhuber, H. Grothe, R. Niessner, U. Pöschl, Raman microspectroscopy of soot and related carbonaceous materials: Spectral analysis and structural information. *Carbon* 2005; 43: 1731-1742. https://doi.org/10.1016/j.carbon.2005.02.018

2. J. Schwan, S. Ulrich, V. Batori, H. Ehrhardt, S. R. P. Silva, Raman spectroscopy on amorphous carbon films. *J. Appl. Phys.* 1996; 80: 440-447. https://doi.org/10.1063/1.362745

3. X. Wang, Y. Hu, L. Song, W. Xing, H. Lu, P. Lv, G. Jie, Flame retardancy and thermal degradation mechanism of epoxy resin composites based on a DOPO substituted organophosphorus oligomer. *Polymer* 2010; 51: 2435-2445. https://doi.org/10.1016/j.polymer.2010.03.053

4. J. Vasiljević, I. Jerman, G. Jakša, J. Alongi, G. Malucelli, M. Zorko, B. Tomšič, B. Simončič, Functionalization of cellulose fibres with DOPO-polysilsesquioxane flame retardant nanocoating. *Cellulose* 2015; 22: 1893-1910. https://doi.org/10.1007/s10570-015-0599-x

5. B.-W. Liu, L. Chen, D.-M. Guo, X.-F. Liu, Y.-F. Lei, X.-M. Ding, Y.-Z. Wang, Fire-Safe Polyesters Enabled by End-Group Capturing Chemistry. *Angew. Chem., Int. Ed.* 2019; 58: 9188-9193. https://doi.org/10.1002/anie.201900356

6. Q.-Z. Wang, C. Liu, Y.-J. Xu, Y. Liu, P. Zhu, Y.-Z. Wang, Highly efficient flame retardation of polyester fabrics via novel DOPO-modified sol-gel coatings. *Polymer* 2021; 226: 123761. https://doi.org/10.1016/j.polymer.2021.123761

7. A.-N. Zhang, H.-B. Zhao, J.-B. Cheng, M.-E. Li, S.-L. Li, M. Cao, Y.-Z. Wang, Construction of durable eco-friendly biomass-based flame-retardant coating for cotton fabrics. *Chem. Eng. J.* 2021; 410: 128361. https://doi.org/10.1016/j.cej.2020.128361

8. C. Fu, Z. Wang, Y. Gao, J. Zhao, Y. Liu, X. Zhou, R. Qin, Y. Pang, B. Hu, Y. Zhang, S. Nan, J. Zhang, X. Zhang, P. Yang, Sustainable polymer coating for stainproof fabrics. *Nat. Sustain.* 2023; 6: 984-994. https://doi.org/10.1038/s41893-023-01121-9

9. F. Ding, J. Liu, S. Zeng, Y. Xia, K. M. Wells, M.-P. Nieh, L. Sun, Biomimetic nanocoatings with exceptional mechanical, barrier, and flame-retardant properties from large-scale one-step coassembly. *Sci. Adv.* 2017; 3: e1701212. DOI: 10.1126/sciadv.1701212

10. M. Wu, B. Ma, T. Pan, S. Chen, J. Sun, Silver-Nanoparticle-Colored Cotton Fabrics with Tunable Colors and Durable Antibacterial and Self-Healing Superhydrophobic Properties. *Adv. Funct. Mater.* 2016; 26: 569-576. https://doi.org/10.1002/adfm.201504197

11. S. Chen, X. Li, Y. Li, J. Sun, Intumescent Flame-Retardant and Self-Healing Superhydrophobic Coatings on Cotton Fabric. *ACS Nano* 2015; 9: 4070-4076. https://doi.org/10.1021/acsnano.5b00121

12. Y. Wang, G. Xia, H. Yu, B. Qian, Y. H. Cheung, L. H. Wong, J. H. Xin, Mussel-Inspired Design of a Self-Adhesive Agent for Durable Moisture Management and Bacterial Inhibition on PET Fabric. *Adv. Mater.* 2021; 33: 2100140. https://doi.org/10.1002/adma.202100140

13. J. Qian, Q. Dong, K. Chun, D. Zhu, X. Zhang, Y. Mao, J. N. Culver, S. Tai, J. R. German, D. P. Dean, J. T. Miller, L. Wang, T. Wu, T. Li, A. H. Brozena, R. M. Briber, D. K. Milton, W. E. Bentley, L. Hu, Highly stable, antiviral, antibacterial cotton textiles via molecular engineering. *Nat. Nanotechnol.* 2023; 18: 168-176. https://doi.org/10.1038/s41565-022-01278-y

14. Z. Miao, D. Yan, T. Zhang, F. Yang, S. Zhang, W. Liu, Z. Wu, High-Efficiency Flame Retardants of a P-N-Rich Polyphosphazene Elastomer Nanocoating on Cotton Fabric. *ACS Appl. Mater. Inter.* 2021; 13: 32094-32105. https://doi.org/10.1021/acsami.1c05884

15. N. Li, H. Han, M. Li, W. Qiu, Q. Wang, X. Qi, Y. He, X. Wang, L. Liu, J. Yu, F. Li, D. Wu, Eco-friendly and intrinsic nanogels for durable flame retardant and antibacterial properties. *Chem. Eng. J.* 2021; 415: 129008. https://doi.org/10.1016/j.cej.2021.129008

16. H. Liu, L. Guo, S. Hu, F. Peng, X. Zhang, H. Yang, X. Sui, Y. Dai, P. Zhou, H. Qi, Scalable Fabrication of Highly Breathable Cotton Textiles with Stable Fluorescent, Antibacterial, Hydrophobic, and UV-Blocking Performance. *ACS Appl. Mater. Inter.* 2022; 14: 34049-34058. https://doi.org/10.1021/acsami.2c07670

17. P. Qi, Y. Li, Y. Yao, J. Sun, L. Li, J. Liu, X. Gu, H. Li, S. Zhang, Ultra washing durable flame retardant coating for cotton fabric by the covalent bonding and interface polymerization. *Chem. Eng. J.* 2023; 452: 139453. https://doi.org/10.1016/j.cej.2022.139453
